# Characterization of the human head louse nit sheath reveals proteins with adhesive property that show no resemblance to known proteins

**DOI:** 10.1038/s41598-018-36913-z

**Published:** 2019-01-10

**Authors:** Jeong Kuk Park, Yu Jin Han, Jae Ho Lee, Sang-Woo Joo, Ju Hyeon Kim, Si Hyeock Lee, SangYoun Park

**Affiliations:** 1 School of Systems Biomedical Science, Soongsil University, Seoul, 06978 Republic of Korea; 2 Entomology Division, Department of Agricultural Biotechnology, College of Agriculture and Life Science, Seoul National University, Seoul, 08826 Republic of Korea; 3 Department of Chemistry, Soongsil University, Seoul, 06978 Republic of Korea; 4 Research Institute of Agriculture and Life Sciences, Seoul National University, Seoul, 08826 Republic of Korea

## Abstract

Human head and body lice attach their eggs respectively to human hair or clothing by female lice secreted glue that hardens into a nit sheath that protects the egg. In this study, a series of experiments were conducted to characterize the glue-like material of the nit sheath. Fourier transform infrared spectroscopy on embryo-cleared nit showed proteinaceous amide I bands. With this result, we determined the amino acid composition of the nit sheath proteins and performed similarity search against the protein products of the body louse genome to identify the candidate nit sheath proteins. The identified two homologous proteins newly named as louse nit sheath protein (LNSP) 1 and LNSP2 are composed of three domains of characteristic repeating sequences. The N-terminal and middle domains consist of tandem two-residue repeats of Gln-Ala and Gly-Ala, respectively, which are expected to fold into β-strands and may further stack into β-sheets, whereas the C-terminal domain contains multiple consecutive Gln residues. Temporal and spatial transcription profiling demonstrated that both *LNSP1* and *LNSP2* are most predominantly expressed in the accessory gland of females of egg-laying stage, supporting that they indeed encode the nit sheath proteins. Further adhesive property of recombinant partial LNSP1 suggests that both LNSP1 and LNSP2 may act as glues.

## Introduction

Louse is a common name for wingless insects in the order Phthiraptera that contains ~5,000 species of obligate parasites living externally on warm-blooded hosts such as birds and mammals^1^. Humans are host to the two closely related lice; the head louse (*Pediculus humanus capitis*) and the body louse (*P*. *humanus humanus*, also referred to as *P*. *h*. *corporis*), as well as to a distant species of the pubic louse (also referred to as crab louse, *Pthirus pubis*), which all feed exclusively on human blood^2–7^. While the pubic louse is taxonomically distant from head and body lice, head and body lice are conspecific and hence indistinguishable if it was not for slight differences in their size, location of infestations, and feeding behaviors. Among these three types of lice, only the body louse is the known vector of bacterial diseases such as the epidemic typhus, trench fever and relapsing fever. Genetic studies on the head and body lice suggest that they diverged 30,000–110,000 years ago as humans began to wear clothing^8–10^. The whole genome sequence of human body louse, which spans 108 Mb (10,773 protein-encoding genes), was first determined using the Sanger method and further comparisons of the transcriptional profiles of head and body lice indicated that few differences exist between the two suggesting that they are conspecific^11–13^. Years later, the whole genome sequence of the human head louse also has been determined using next generation sequencing^14^.

The ancestral human head louse prefers to attach its eggs to scalp hair rather than to clothing as is the case with the body louse. An adult female head louse, in particular, lays up to ~10 eggs per day, and the ~0.8 mm oval-shaped eggs are attached firmly to the hair by a glue-like material that the female louse secretes from the accessory gland (also known as the glue gland or the collateral gland). This glue quickly hardens into a sheath to cover and protect the egg, while the embryo breathes through a glueless dome called an operculum. After 6–9 days, the egg hatches, and the louse nymph leaves behind its egg shell (usually known as the “nit”). The nit may remain attached to hair for a long time if not physically removed. Current treatments for head louse infestations are limited to topical applications of insecticides, benzyl alcohol or silicone oils, followed by combing with a fine-tooth comb. Moreover, manual removal of eggs at some point is a necessary step for total eradication because the above treatments are not 100% effective in killing the embryo inside the egg. To date, no known compound removes 100% of the eggs/nits from the hair^15,16^.

During the course of evolution, the nit sheath undoubtedly has been optimized and tailored to tenaciously attach the live eggs to either human hair or to the clothing, and has succeeded in becoming one of nature’s high performance glues. The molecular composition of the nit sheath was previously thought to be chitin-based, but more recent studies of the nits using histochemical analysis and flash pyrolysis gas chromatography/mass spectrometry support that they are of protein origin^17–19^. In this study, we sought to identify the protein nature of the louse nit sheath by analyzing the amino acid composition of the nits and then using these results in a similarity search against previously deduced proteins determined from the body louse genome as a reference. Temporal and spatial transcription profiling demonstrated that the identified genes are only expressed in the accessory gland of females at the egg-laying stage, supporting that they most likely encode the nit sheath proteins. Further, adhesive property of the recombinantly expressed gene product suggested that the gene-encoded proteins may likely function as glues.

## Results

### Dissolving the embryos for viable eggs

Head louse eggs were treated overnight with 12 M urea, 74 mM Tris base, and 78 mM dithiothreitol (DTT), which have been used to dissolve and study animal keratin^20^. When the urea-treated eggs were observed under the microscope, they were devoid of the developing embryos (Fig. S1). Furthermore, the clear pale-brownish supernatant of the urea-treated eggs contained proteins of louse origin, indicating that the treatment successfully dissolved the embryos (Table S1). Nevertheless, all attempts to dissolve the remaining nit sheath using organic solvents, such as DMSO, ethanol, and cyclohexane or detergents such as sodium dodecyl sulfate (SDS), Triton^TM^ X-100, and *N*,*N*-dimethyldodecylamine *N*-oxide, failed. Thus, the insolubility of the nit sheath deterred the SDS-PAGE analysis of the nit sheath protein, and the relatively straightforward method of protein identification using mass spectrometry.

### Infrared micro-spectroscopic analysis of the head louse nit

**Figure 1:**
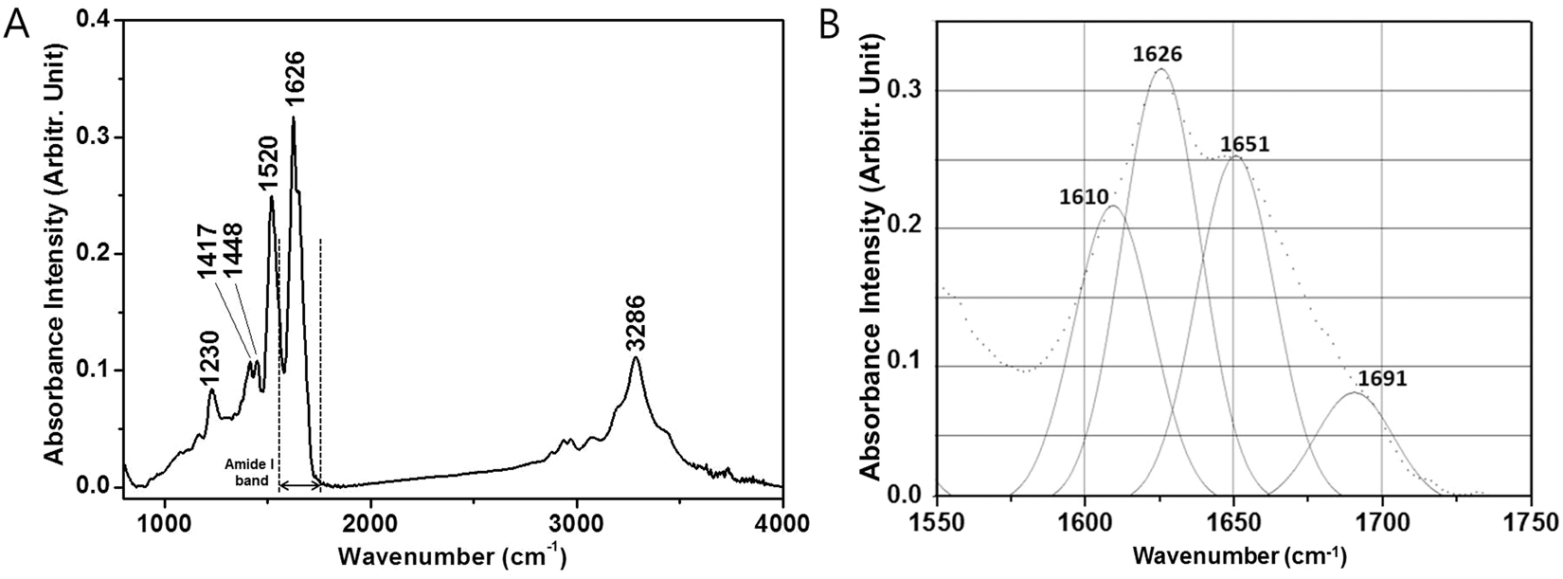
FTIR analysis of the head louse nit. (**A**) FTIR micro-spectroscopic analysis was performed on a single nit devoid of the embryo, which supported that all the peaks are of protein origin. (**B**) The deconvoluted amide I bands are centered around 1610 cm^−1^, 1626 cm^−1^, 1651 cm^−1^, and 1691 cm^−1^, however they could not be used to determine the exact secondary structure content of the nit protein.

Alternatively, Fourier transform infrared (FTIR) spectroscopy is an established tool for secondary structure characterization of proteins, and has an advantage in using solid samples. Thus, an FTIR micro-spectroscopic analysis was performed on a single nit that was devoid of the developing embryo (embryo-cleared nit) to determine the secondary structure states of the proteins in the nit. The spectroscopic result indicated that all the infrared bands were of protein origin^21^ (Fig. 1A) with amide I peaks of 1610 cm^−1^, 1626 cm^−1^, 1651 cm^−1^, and 1691 cm^−1^ (Fig. 1B). Unfortunately, the exact secondary structure content of the nit, expected to be mixtures of α-helices, β-strands and even denatured aggregates, were undeterminable due to the complexity and the overlap of peaks representing certain secondary structure elements in the spectrum.

### Amino acid composition analysis of the head louse nit

**Figure 2:**
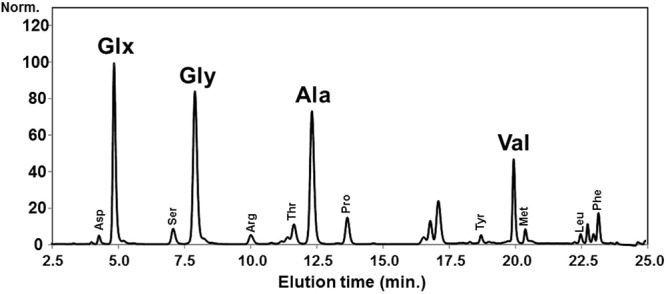
Amino acid composition analysis of the head louse nits. The amino acid composition analysis was performed on the nits that are devoid of the embryos. The elution volumes and the areas under the amino acid peaks of the sample chromatogram were compared to that of the standards to determine the amino acid types and the relative mole percentage of the amino acids composing the nit. The contents of cysteine and tryptophan were undetermined due to the harsh HCl hydrolysis condition.

Since the insolubility of the nit sheath deterred using mass spectrometry for protein identification, the tissue-cleared nits were subjected to amino acid composition analysis by HCl hydrolysis, phenylisothiocyanate (PITC)-derivatization of the amino acids, and subsequent HPLC analysis (Figs 2 and S2). The result indicated that the nit sheath consists mostly of Gly (25.4%), Glx (Glu or Gln, 24.4%), Ala (20.2%) and Val (10.0%) residues (Table 1). The contents of cysteine and tryptophan amino acids could not be determined due to the harsh HCl hydrolysis condition, which degrades these amino acids.

**Table 1:** Experimentally determined amino acid composition of the head louse nit sheath.

Amino acid	% (in moles)
Asx (Asp or Asn)	1.1
Glx (Glu or Gln)	24.4
Ser	2.5
Gly	25.4
His	N.D.*
Arg	1.7
Thr	3.5
Ala	20.2
Pro	3.8
Tyr	0.8
Val	10.0
Met	1.1
Ile	N.D.*
Leu	0.9
Phe	4.7
Lys	N.D.*

*Non-detected.

### Bioinformatic search for candidate nit sheath proteins using the body louse genome

**Figure 3:**
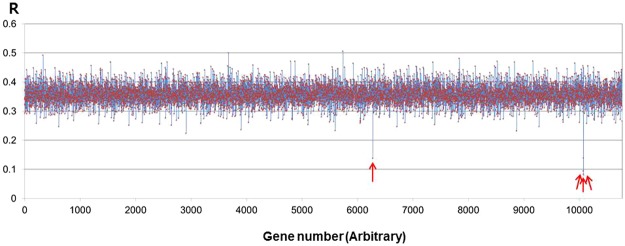
Bioinformatic search of candidate protein. Root sum squares (R) of the 18 amino acid (excluding cysteine and tryptophan) offsets between the amino acids of 10,773 proteins encoded by body louse genome and the amino acid analysis result of the nit sheath were calculated. The R values ranged from 0.083 to 0.506, and the four candidate proteins with lowest R (shown in arrows) were identified as the candidate proteins of the nit. The values on the horizontal axis are from the order of body louse genes listed in NCBI.

Due to the incompleteness of the human head louse genome sequence, the whole genome of human body louse was used as the reference for our bioinformatic search for the identification of candidate proteins that comprise the head louse nit sheath. The sequence-derived compositions using 18 amino acids of all protein products deduced from the body louse genome were compared with the experimental contents resulting from the amino acid composition analysis of the tissue-cleared nit. A root sum squares (R) of the offsets between the 18 amino acids (excluding cysteine and tryptophan) of the 10,773 human body louse proteins and the experimental values of the nit amino acid composition were calculated where the R-values ranged from 0.083 to 0.506 (Fig. 3). From this analysis, the candidate proteins of the louse nit sheath were narrowed down to the four gene products of PHUM596000 (R = 0.083), PHUM595880 (R = 0.095), PHUM403440 (R = 0.138), and PHUM595890 (R = 0.139) (Fig. 3 and Table S2). Other louse proteins with R-values greater than 0.2 showed significant discrepancies in the contents of Gln, Gly, Ala and Val residues. Of these four candidate genes of the nit sheath, PHUM596000, PHUM595880, PHUM595890 are reported to be complete sequences whereas PHUM403440 is an incomplete partial sequence. In genomic context, PHUM595880 and PHUM596000 are present in a tandem array whereas PHUM595890 is positioned in the reverse orientation between PHUM595880 and PHUM596000. Close inspection of PHUM595890 indicated multiple undetermined bases in the original shotgun reference sequence. PHUM596000, PHUM595880, and PHUM595890 are expected to encode proteins of 569, 434 and 127 amino acids respectively (Table S2). Being a partial sequence, the location of the PHUM403440 in the body louse genome has not been determined. The partial sequence available for PHUM403440 is expected to encode a protein of at least 169 amino acids (Table S2). With respect to the high Gln content found in the amino acid analysis, all the protein products of the four genes had several polyglutamine (polyQ) regions where tandem consecutive Gln residues are seen. The functions of these four genes, however, are all unknown and hence have been annotated as hypothetical proteins. NCBI-BLAST protein searches using these protein sequences showed no similar protein from other organisms in database.

### DNA amplifications and sequence analysis of the candidate nit sheath genes using the body and head lice cDNA

In order to confirm that the candidate nit sheath genes are actually transcribed into mRNA in body louse, PCR amplifications of the four genes were performed using the body louse cDNA generated from female lice mRNA. Although the relatively small-sized PHUM595890 and PHUM403440 failed to amplify, the DNAs of PHUM595880 and PHUM596000 were obtained (Fig. S3), strongly suggesting that PHUM595880 and PHUM596000 are proteins encoded by genes of human body lice. Because the whole genome sequence of head louse is also known, the head louse homologs of the four candidate nit sheath genes were searched and those for PHUM403440, PHUM595880 and PHUM596000 found (Fig. S4). The PCR products of PHUM595880 and PHUM596000 in both head and body lice were successfully sequenced using the Sanger method. The sequencing results also confirmed that there are no introns in the PHUM595880 and PHUM596000 genes. Interestingly, our overall nucleotide sequence of body louse PHUM595880 suggested that its protein sequence is 100% identical to the partial sequence of body louse PHUM403440 (Fig. S4A). Also, the protein product of head louse PHUM595880 is 95.8% identical to that of the head louse PHUM403440 at the aligned regions (Fig. S4B). Moreover, the protein product of body louse PHUM595890 with a total of 127 amino acids (Table S2) differs from body louse PHUM596000 mostly due to alterations in the N-terminal 37 residues that harbor the signal sequence (Fig. S4C). Other than this, it is nearly identical to PHUM596000 with only two residues out of the remaining 90 residues in C-terminal region differing (Fig. S4C). For these reasons, we regard that PHUM403440 and PHUM595890 are partial copies of PHUM595880 and PHUM596000, respectively, and only analyze the gene products of PHUM595880 and PHUM596000 hereafter calling them louse nit sheath protein (LNSP) 1 and LNSP2, respectively.

### Comparative molecular characterization of deduced amino acid sequences of head and body lice LNSP1/2

**Figure 4:**
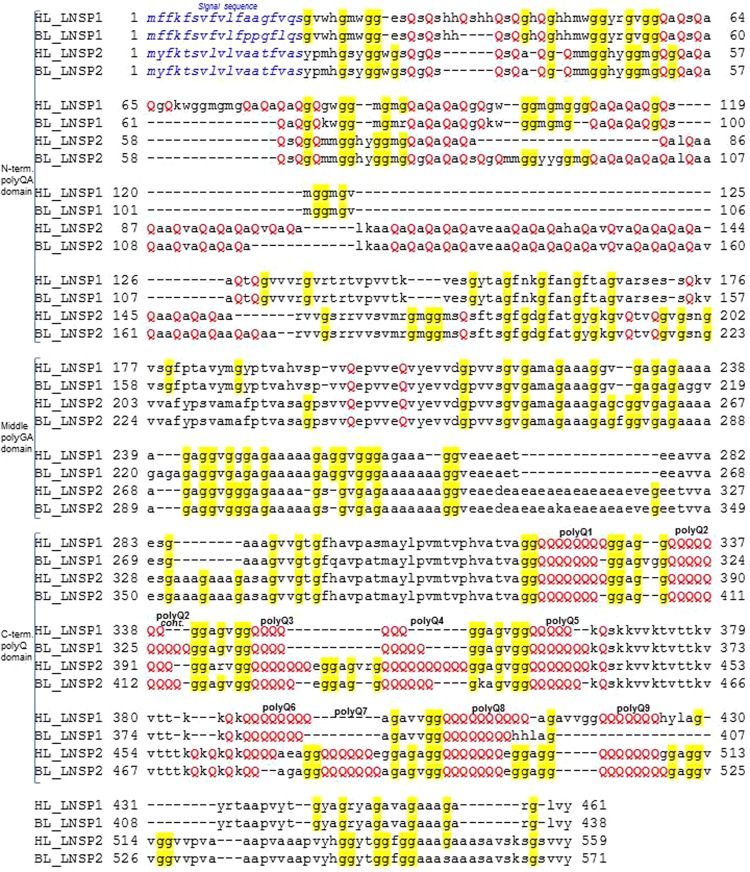
Protein sequence alignment of candidate louse nit sheath proteins (LNSP1 for protein product of PHUM595880, and LNSP2 for protein product of PHUM596000). The LNSP1 and LNSP2 are 69% identical in deduced amino acid sequence. Both proteins contain the N-terminal sequences that are predicted as signal sequences (colored in blue). Subsequent regions can be divided into three domains each holding characteristic repeat sequences, which are the polyQA sequence (N-terminal domain), the polyGA sequence (middle domain), and the polyQ sequence (C-terminal domain). Conserved residues of Gln (capitalized in *red*) and Gly (in *yellow* background) are shown. The protein sequences are translated from our DNA sequencing results.

The overall amino acid sequences of head and body lice LNSP1/2 deduced from the Sanger sequencing were aligned for comparative characterization of the proteins (Fig. 4). The N-terminal regions of 18 amino acids in all four proteins are predicted as signal sequences. Subsequent regions of the proteins can be divided into three domains which hold characteristic repeating sequences (Figs 4 and S5). It is noteworthy that the three domains are connected by non-repeating sequences that are also conserved between LNSP1 and LNSP2, and that all three domains are more elongated in LNSP2 than in LNSP1 (Fig. 4).

The N-terminal domain is represented by the polyQA (also partly QS) sequence and the middle domain by the polyGA (also partly GV, GS or GG) sequence. The characteristic two-residue repeats of these two domains are indicative of a β-strand folding that may further pack laterally into interacting β-sheets. In the N-terminal domain, the alternating Gln-Ala (or Ser) residues would place the side chains of Gln on one side and Ala (or Ser) on the other. When the four protein sequences of head and body lice LNSP1/2 are compared, the N-terminal polyQA domain holds interesting polymorphisms of insertions and deletions (i.e. only head louse LNSP1 has an extra 15-residue insertion and head louse LNSP2 has a deletion of 21-residue, Fig. 4). In general, more elongated repetitions of QA sequences (65–70 residues) for only LNSP2 lead to larger LNSP2 size compared to LNSP1.

Similar to the N-terminal polyQA domain, the two-residue repeats in the middle polyGA domain would place the small hydrogen side chains of Gly on one side with Ala (also Val, Ser or Gly) projecting on the other. Such repeating sequences would allow efficient interaction between the β-strands as observed in the Gly-Ala or Gly-Ser repeats of insect and spider silk fibroins. Also in this domain, two regions of 4–7 Ala residues in tandem exist. After this region, an insertion of 24-residue is only observed in LNSP2.

In contrast to the two-residue repeats of the N-terminal and middle domains, multiple polyQ sequences of 5–10 Gln residues in tandem are found in the C-terminal domain. The aligned sequences of the four proteins suggest at most nine of these polyQ regions (termed polyQ1–Q9) in LNSP1 and LNSP2 (Fig. 4). Most dramatic differences between LNSP1 and LNSP2 takes place in this C-terminal polyQ domain. For instance, the exact numbers of Gln residue in these polyQ regions are not conserved between LNSP1 and LNSP2. Also, polyQ3 and polyQ4 regions of LNSP2 are combined into one in LNSP1, and polyQ7 is only seen in LNSP2. More interestingly, regions harboring polyQ9 is deleted in only body louse LNSP1.

Head louse LNSP1 and LNSP2 show 68% identity in their overlapping amino acid sequence, which is similar to the 69% identity observed for that of the body louse LNSP1 and LNSP2 (Fig. 4). However, considerable differences in the amino acid sequences of LNSP1 (Fig. S6A) and LNSP2 (Fig. S6B) were observed between the body and head lice. The sequence variations between head and body lice LNSP1s (or LNSP2s) resulted in 3–4% differences in amino acids (For detailed comparisons refer to SI Results and Fig. S6). Moreover, some single residue variants as well as deletion and insertion changes were identified in body louse LNSP1 and LNSP2 when our sequence of the San Francisco (BL_SaFr) strain and the previously reported 2010 sequence^12^ of the Culpepper (BL_Culp) strain were compared (as refer to SI Results and Fig. S6). The results indicated high levels of polymorphisms in the two proteins. Overall, 1.6% of amino acids were different in both LNSP1 and LNSP2 between the BL_Culp sequence and our BL_SaFr sequence. Hence the inter-subspecies sequence variations (3–4%) between head and body lice LNSP1s (or LNSP2s) were higher than the intra-subspecies differences (1.6%) for the polymorphisms between LNSP1s (and LNSP2s) of the two body louse strains (BL_Culp vs. BL_SaFr). In any case, these overall changes in the two proteins had a small impact on the amino acid content determined; hence the previous bioinformatic search is still valid.

### Accessory gland-specific expression of LNSP1 and LNSP2 in head louse gravid females

**Figure 5:**
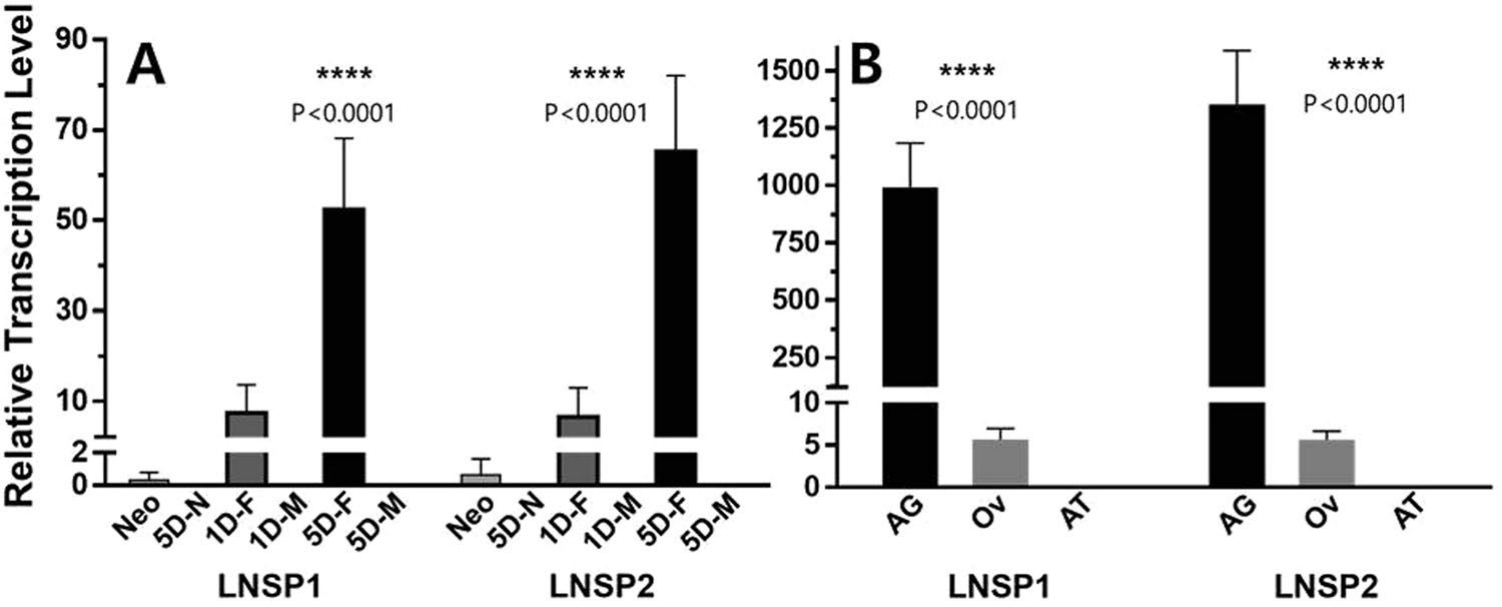
Temporal and spatial transcription profiles of *LNSP1* and *LNSP2* in various developmental stages (**A**) and different female organs (**B**) of head lice. The ****mark indicates the statistically significant (p < 0.0001) mean value as judged by one-way ANOVA in conjunction with Tukey’s test. Abbreviations in the horizontal axis represent the following: Neo, neonate; 5D-N, 5-day old nymph; 1D-F, 1-day old female; 1D-M, 1-day old male; 5D-F, 5-day old female; 5D-M, 5-day old male; AG, accessory gland, Ov, ovary; AT, alimentary tract. The transcription levels of both LNSP1 and LNSP2 in 5D-N, 1D-M, 5D-M and AT were too low (0.005~0.022) to be seen in the graph.

To quantify the transcription levels of *LNSP1* and *LNSP2* in different developmental stages of head louse, qPCR experiments were conducted (Fig. 5A). Both *LNSP1* and *LNSP2* were most predominantly transcribed in the 5-day old females, and then followed by the 1-day old females, neonates, 1-day old males, 5-day old males and 5-day old nymphs. The relative transcription levels of *LNSP1* and *LNSP2* in the 5-day old females were 5,900- and 13,800-fold higher compared to those in the 5-day old nymphs, respectively. When the transcription levels in the 5-day old females were compared with those in males, the fold differences were more than 3 orders of magnitude for both *LNSP1* and *LNSP2*. In addition, the 5-day old gravid female exhibited significantly higher transcription levels of *LNSP1* and *LNSP2* (7.1 and 10.1 fold, respectively, p < 0.0001, ANOVA in conjunction with Tukey’s test) compared to the 1-day old female, suggesting that the expression of *LNSP1* and *LNSP2* is specifically associated with egg-laying stage. The transcription levels of *LNSP1* and *LNSP2* in the 5-day old females were almost identical (p = 0.565, ANOVA in conjunction with Tukey’s test). Interestingly, the overall transcription levels of *LNSP1* and *LNSP2* in the 5-day old female were 52–65 fold higher compared to that of the *actin-5c*, an internal reference gene typically showing a high expression level, suggesting that these two genes are expressed in large amounts during the oviposition period.

To identify the tissues expressing LNSP1 and LNSP2 in gravid females, the spatial transcription levels of *LNSP1* and *LNSP2* in the accessory gland, ovary and alimentary tract were determined by qPCR (Fig. 5B). Both *LNSP1* and *LNSP2* were exclusively transcribed in the accessory gland. Only low levels of transcription were observed in the ovary. Transcription levels of *LNSP1* and *LNSP2* in the accessory gland were 170- and 240-fold higher compared with those in the ovary, respectively (p < 0.0001, ANOVA in conjunction with Tukey’s test). Little detectable level of transcription was observed in the alimentary track (0.011 and 0.004 relative transcription levels for *LNSP1* and *LNSP2*, respectively). These findings demonstrate that both LNSP1 and LNSP2 are specifically expressed in the accessory gland, and thus constitute a major part of the nit sheath protein.

### Recombinant expression of partial body louse LNSP1 and its characterization

**Figure 6:**
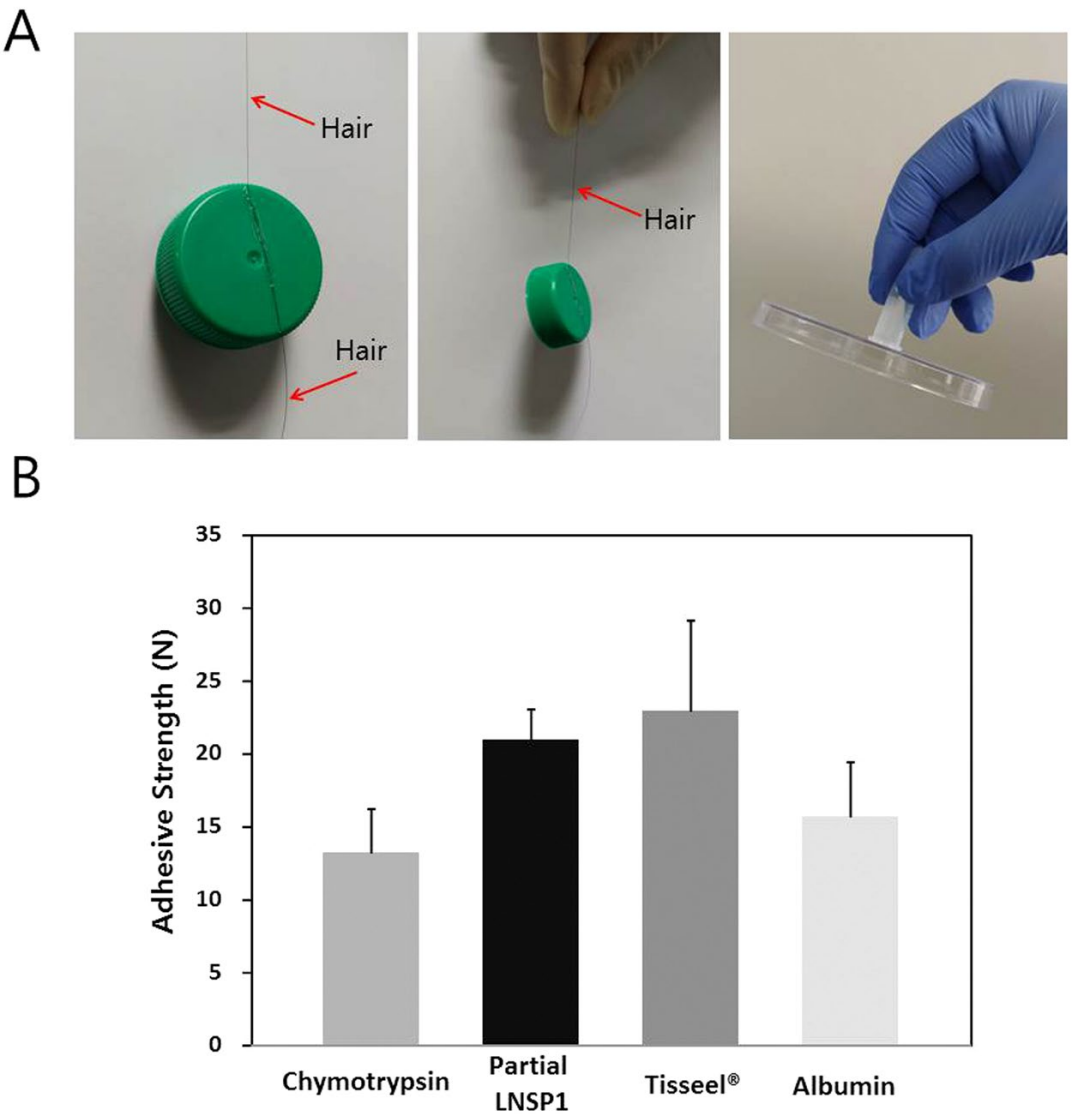
Adhesive property of LNSP1. (**A**) Partial LNSP1 was recombinantly expressed in *E*. *coli* and was used to glue human hair onto plastic, or stick together laboratory plastic wares. (**B**) The forces needed to break the polypropylene films attached together using various proteins were measured using a universal testing machine (UTM). The partial LNSP1 showed as much adhesive property as the commercially available fibrin-based glue (Tisseel®) even at a ~500-fold (in grams) less amount (See SI Methods). Chymotrypsin and BSA showed weaker binding than the partial LNSP1. The error bars are for one standard deviation of at least three repeated experiments.

In order to characterize the function of our identified nit protein, gene encoding body louse LNSP1 was cloned into an *E*. *coli* expression vector, and tested for expression as a water soluble form. While the full LNSP1 (residues 19–438) without the putative signal sequence (residues 1–18) remained within the bacterial inclusion body during expression and hence failed to be purified as a soluble form (results not shown), a partial LNSP1 without the polyQ C-terminal domain (residues 19–303 containing only the N-terminal and the middle domains) was obtainable in a soluble form. Although the protein was soluble initially at low concentration, it turned into a tacky state with concentration during the purification step. This concentrated partial LNSP1 solidified into a thin film upon evaporation of water when applied on solid surface, and was used to glue human hair onto plastic, or stick together laboratory plastic wares (Fig. 6A). Moreover, the partial LNSP1 was also tested for adhesive strength on polypropylene (PP) films, which was measured using a universal testing machine (UTM) to pull apart the glued PP films. The adhesive property of the partial LNSP1 was compared to chymotrypsin, bovine serum albumin (BSA), and the commercially available fibrin-based biological adhesive (Tisseel®). The partial LNSP1 showed stronger adhesive property than the chymotrypsin and BSA (Fig. 6B). Also, the partial LNSP1’s adhesive property was comparable to fibrin even when the applied amount on the PP film was ~500-fold (in grams) less.

## Discussion

Although we attempted to determine the secondary structure of the embryo-cleared nit using FTIR micro-spectroscopy, it only confirmed that the nits are of protein origin. However, the amino acid composition of the head louse nit sheath was used to identify candidate nit sheath proteins by searching it against the body louse whole genome database. A straightforward way of protein identification by using mass spectrometry was not feasible because the nit sheath failed to dissolve under all the conditions tested. In a previous study performed on the nit sheath of head lice, a treatment of 2.7 M urea, 3.3% SDS, 0.3 M Tris pH 6.8, and 3.3% β-mercaptoethanol was reported to dissolve the nit sheath^22^. However, our eggs that had been treated to remove the embryos failed to dissolve under the same condition.

Our amino acid composition result determined that the nit sheath protein consisted mostly of Gly (25.4%), Glx (24.4%), Ala (20.2%) and Val (10.0%) residues. In a previous study, the putative glue extract was extracted from the accessory gland of body lice using hydrochloric acid, chloroform, and methanol^23^. Subsequent amino acid composition analysis was performed on the five major SDS-PAGE protein bands of the glue extracts and showed similar composition in all of the five bands with average of Gly (23.1%), Glx (17.8%), Ala (9.8%) and Val (8.3%)^22^. Previous result also suggested substantial amounts of Asx (13.0%), Leu (8.4%), and Lys (7.4%); however, our analysis indicated only minimal contributions (at most 1%) of those residues. Because the proteins of two louse subspecies - head and body lice - are not expected to differ to such extent, the different amino acid composition results from the two studies are not explainable at this time.

When our amino acid composition of the head louse nit sheath was compared against all of the gene products of the body louse, four deduced protein products from the PHUM403440, PHUM595880 (named as LNSP1 in this study), PHUM595890 and PHUM596000 (named as LNSP2 in this study) genes had the highest content matches, suggesting them as likely nit sheath proteins. The tandem array of *LNSP1* and *LNSP2* in the body louse genome suggests that they were generated from a common ancestral gene *via* a relatively recent gene duplication event. As the head louse is the ancestor of the body louse, the same tandem array of two genes is expected in the head louse genome as well. Along with *LNSP1* and *LNSP2*, presence of partial related genes (PHUM403440, *LNSP1-like*; PHUM595890, *LNSP2-like*) in the genomes of both head and body lice suggests that there had been at least two rounds of *LNSP* gene duplication during evolution.

No matches to other known egg shell proteins of insects further support their identity as nit sheath (or glue) protein rather than any protein components forming egg shell. Previous analysis of the body louse genome indicated that 163 protein products out of the total 10,773 protein-coding genes show no known orthologues in other well-studied organisms^11–13^, and all four proteins were found to be unique to human lice. It is worth noting that for the bioinformatic search, we used the genome sequences of body louse as a reference genome rather than using that of the head louse because the whole genome sequences of human head louse only covered 96% of the body louse genome^14^. The incompleteness of the head louse genome was suggested to be, in part, from the inaccurate assembly of the highly variable sequences obtained from the heterogeneous head louse genomic DNA^14^. Nevertheless, intact genes that are orthologous to body louse *LNSP1* and *LNSP2* were identified from the head louse genome and their sequences were determined to be highly similar to those of the body louse (*see below*). In general, analysis of the amino acid content has a limitation that total hydrolyzed amino acids of minor proteins that compose the nit sheath are buried within those of the major proteins. Hence, despite the fact that LNSP1 and LNSP2 are likely the major component of the nit sheath, the actual nit sheath may be composed of other minor proteins as well along with LNSP1 and LNSP2 into a complex aggregated mixture.

A previous study by Carter^23^ analyzed the body louse glue proteins isolated from accessory glands. In the study, five major proteins were observed with molecular masses of the four largest proteins reported as ~42 kDa, ~52 kDa, ~55.5 kDa and ~69 kDa, while the size of the fifth protein could not be accurately estimated because it eluted near the SDS-PAGE gel front. Also in a previous study using head lice nit sheath^22^, four proteins with molecular masses of ~50 kDa for two, and ~20 kDa for the other two were observed. It is not possible, however, to make any reasonable comparisons of theses sizes to the molecular masses of our candidate nit sheath proteins [PHUM595880 (LNSP1, ~43 kDa), PHUM595890 (LNSP2, ~13 kDa), and PHUM596000 (LNSP2, ~55 kDa)] in that the secreted nit sheath proteins can have increased apparent sizes resulting from post-translational glycosylation.

The two genes, *LNSP1* and *LNSP2*, in the present study were PCR-amplified from cDNA of both head and body lice, which supports the contention that these genes are actually expressed as proteins in lice. Previous comparisons between head and body lice nucleotides show low (0.1–1.3%) overall diversity in majority of transcripts^13^, and on average 2.2% (up to 8.1%) diversity in the genomic single nucleotide polymorphisms^14^. However, our results indicated that significant amino acid sequence difference (3–4%) exists within *LNSP1* (or *LNSP2*) between head and body lice, and even 1.6% amino acid sequence difference takes place within *LNSP1* (or *LNSP2*) of the two body lice sequences from different strains. Given the fact that not all single nucleotide polymorphisms induce amino acid changes and the average inter-subspecies nucleotide differences being 0.1–1.3%, the 3–4% amino acid difference between *LNSP1* (and *LNSP2*) of head and body lice is exceptionally high, which may be accumulated by high rates of mutation in the *LNSP1* and *LNSP2* genes. Perhaps the rapid rates of evolution particularly in lice LNSP1 and LNSP2 may have been necessary to adapt to the change of egg-laying environment from hair shaft (i.e., head lice) to fabric (i.e., body lice), thereby enhancing the overall survivorship of lice offspring.

Both LNSP1 and LNSP2 have predicted signal sequences at the N-terminus. These sequences of consecutive hydrophobic residues indicate that the proteins are likely to be secreted. Based on repeating motifs, the remaining regions of LNSP1 and LNSP2 can be divided into three domains. The mostly polyQA N-terminal domain and the polyGA middle domain are expected to fold into β-strands, which project Gln and Gly on one side and Ala on the other. The sequence of the polyGA middle domain is somewhat similar to silk fibroin, which contains mostly the smallest amino acids such as Gly, Ser, and Ala that folds into a stacking antiparallel β-sheet^24^.

The C-terminal polyQ domain is represented by multiple polyQ sequences of 5–10 Gln residues in tandem. PolyQ sequences in proteins have been implicated in many neurodegenerative diseases, including Huntington’s disease^25–28^. In such cases, the neurologic disorders are caused by an increased number of CAG repeats from a non-coding CAG trinucleotide microsatellites inserted into the coding regions of proteins. These expanded CAG repeats are found translated into a series of uninterrupted polyQ repeats that mediate problematic aggregation of the proteins. From structure databases, the structure of Huntingtin protein which is linked to the Huntington’s disease has been shown with 17-tandem Gln at the N-terminus^29^ (PDB code 3IO4). However, the polyQ was inferred as flexible and unstructured with no particular secondary structure. Such unstructured repeats can induce aggregation of proteins. In the course of evolution, the human louse may have utilized the widely occurring non-coding CAG trinucleotide microsatellites in their genome to their advantage in order to create genes such as *LNSP1* and *LNSP2* that may use aggregation for functional benefit.

Temporal and spatial transcription profiling based on qPCR using various developmental stages of the head louse and different female organs demonstrates that both LNSP1 and LNSP2 are exclusively expressed in the accessory gland of egg-laying females. Low levels of transcription in the ovary indicate that neither LNSP1 nor LNSP2 is likely a main constitute of egg shell (nit). Taken together, these findings strongly support that both LNSP1 and LNSP2 constitute main part of nit sheath proteins. The transcription levels of both *LNSP1* and *LNSP2* being almost identical in the accessory gland, suggests that both proteins may be equally essential in making the nit sheath. The fact that both LNSP1 and LNSP2 were transcribed in large amounts, particularly in the accessory gland of gravid females, agrees well with the notion that a large amount of nit sheath proteins is likely required for egg-laying process and is secreted from the accessory gland.

As discussed earlier, previous studies identified 4–5 major proteins on SDS-PAGE, which constitute either the glue proteins from body louse accessory glands or head louse nit sheath^22,23^. If assuming that LNSP1 and LNSP2 are candidates for the 40–60 kDa proteins, the identities of the other proteins remain to be identified. The transcriptome analysis of accessory gland or proteome analysis of accessory gland secretion would provide insights into the complete constitution of louse nit sheath proteins.

The adhesive property of the expressed partial LNSP1 that is manifested as for that of a well-known adhesive fibrin suggests that one likely role of LNSP1 may to work as a glue protein secreted from the accessory gland. Repeated attempts in recombinant expressions of full-length LNSP1 which includes the polyQ domain or even LNSP2 without the polyQ domain using *E*. *coli* were not successful mostly likely attributed from their larger size and hence lowered solubility in comparison to the partial LNSP1 of only the N-terminal and the middle domains. Nevertheless, as the homologous LNSP2 has similar structural motifs as LNSP1, LNSP2 is also suggested to show a glue-like behavior but detailed functional analysis would be necessary to confirm this notion. It is interesting to note that since LNSP1 becomes tacky upon concentration even without the polyQ-containing C-terminal domain, the native form of LNSP1 containing the C-terminal domain may show even greater adhesion property.

High levels of Gly, Ser, Pro, and Gln are typical of elastic and adhesive proteins^30^. In the case of LNSP1 and LNSP2, Gly consists ~20% of the sequence while levels of Ser (4–5%) and Pro (2%) are low. High contents of Gln in LNSP1 (Gln 18%) and LNSP2 (Gln 21%) are unique and exceptional even among known biological adhesives. In other biological adhesives with known amino acid compositions, a defensive glue secreted by the Australian frog *Notaden bennetti* (Glx 14%), and two egg attachment glues of the Australian moth *Opodiphthera eucalypti* (Glx 18%) and the Australian ladybird beetle *Harmonia conformis* (Glx 14%) have been reported to have high Glx content^30–32^.

In order to function as adhesives, glue proteins must exist as liquid within the accessory gland but solidify once on human hair (or clothing). Oxygen exposure has been proposed previously to activate this liquid-to-solid curing process^16^. Based on our adhesive experiment using expressed partial LNSP1 without the polyQ-containing C-terminal domain, it is tempting to speculate that evaporation of water or (and) exposure to oxygen indeed can be the triggering factors for the curing. Because the native LNSP1 and LNSP2 also contain the polyQ C-terminal domain, there can be more layers of complexity during curing as well. For example, since LNSP1 and LNSP2 contain a total of 14 and 15 lysines, respectively, one inferred possibility is the covalent crosslinking taking place between ε-amino group of a lysine side chain and γ-carboxamide group of a Gln side chain mediated by a glutamine γ-glutamyltransferase (or transglutaminase). Formation of multiple lysine-glutamine crosslinkings is well-known to happen during fibrin assembly in the final step of blood clotting^33,34^. Both head and body louse genomes contain one putative glutamine γ-glutamyltransferase gene, and it remains to be seen whether this protein product is secreted from the accessory gland.

Although LNSP1 and LNSP2 may presumably function as adhesive proteins, they may provide other functions, such as preservation of water and defense against pathogenic microorganisms, which remains to be elucidated. Silencing of *LNSP1* and *LNSP2* singly or in combination *via* RNA interference would provide insights into their physiological functions.

## Conclusions

No detailed characterization of the protein(s) making up the nit sheath is available to date. FTIR spectroscopy analysis on the nit sheath shows proteinaceous amide I bands, and we have identified two putative nit sheath proteins, LNSP1 and LNSP2 using amino acid analysis. Temporal and spatial transcription profiling demonstrates that both *LNSP1* and *LNSP2* are only expressed in accessory glands of females in the egg-laying stage, supporting that they most likely encode nit sheath proteins. Further recombinant expression and characterization of partial LNSP1 suggest that perhaps one function of LNSP1 and LNSP2 may be to act as glues. Amino acid sequences of these proteins suggest a tandem two-residue repeats of Gln-Ala and Gly-Ala, respectively, at the N-terminal and the middle domains, which are expected to fold into β-strands that may further stack into β-sheets. Also, multiple appearances of consecutive Gln residues represent the C-terminal domain, which is reminiscent of the polyQ found in problematic proteins associated with neurodegenerative diseases.

Molecular structures and the detailed functions on LNSP1 and LNSP2 remain to be determined. If validated as glue proteins, discovering agents that target LNSP1 or LNSP2 can lead to novel treatments for louse infestations since compounds designed to either inhibit the curing or loosen the solidified glues from the hair shaft or the clothing would hinder the overall attachment of eggs^6,16^. Moreover, future studies that reveal the detailed cementing mechanism of louse glue proteins can lead to their development into a high performance biological adhesive as in the example of the waterproof glue derived from the mussel’s byssal thread^35,36^.

## Methods

According to the Korean bioethics and safety act, this research was exempt from ethical approval and the subjects or legal guardians provided informed consents. Human head lice eggs and nits (empty egg cases) were collected from five individuals in a family infested with head lice. The louse embryos inside the egg cases were chemically dissolved and eliminated from the egg case (leaving a nit) using a buffer containing 12 M urea, 74 mM Tris base, and 78 mM dithiothreitol (DTT). The pale-brownish supernatant of the dissolved embryo was analyzed to identify its protein using trypsin digestion and LC-MS/MS. To remove any other contaminating proteins on nits, they were further treated with 4% sodium dodecyl sulfate (SDS) and 5 mM DTT. The morphology of the nits before and after this treatment was identical with the nit sheath remaining undissolved. An IR micro-spectrum of a single head louse nit sheath completely devoid of the embryo was obtained using a FTIR microscope spectrometer and the deconvolution of the amide I bands were performed. Five nits prepared as above were used for the amino acid composition analysis, in which the samples were hydrolyzed using 6 N HCl and the resulting amino acids modified with PITC. A HPLC chromatogram on the mixture was compared to that of the amino acid standards to identify and determine the amino acid type and their relative mole percent. In order to search for the louse protein whose amino acid composition corresponds to the amino acid analysis result of the nit sheath, a root sum squares (R) in the composition offsets of the amino acids between human body louse encoding 10,773 proteins and the experimentally determined louse nit sheath were calculated. To confirm that the genes of the identified candidate nit sheath proteins are actually transcribed in lice, PCR amplifications of the genes were performed using body louse cDNA. Furthermore, the PCR products were fully sequenced using the Sanger method. To quantify the transcription levels of candidate nit sheath protein genes, *LNSP1* and *LNSP2*, in different developmental stages and in different organs (accessory gland, ovary and alimentary track), quantitative real-time PCR (qPCR) was conducted. Relative transcription levels were estimated using *actin-5c* or *RpL13A* as internal reference genes. For recombinant expression of LNSP1, DNA encoding only the N-terminal and middle domains of LNSP1 (without the putative signal sequence) (19–303 of full-length 1–438) were cloned into a bacterial expression vector. The partial LNSP1 with N-terminal His_6_-tag was over-produced with soluble expression of the protein, and further purified using affinity and size-exclusion chromatography. The partial LNSP1 was used to glue PP films and adhesive strength measured using a universal testing machine. Details on the experimental procedures can be found in SI Materials and Methods^37–39^.

## Supplementary information

SI Results, SI Methods, and SI Figures

## Data Availability

The sequences reported in this paper have been deposited in the GenBank database (“GenBank: MF988352” for body louse LNSP1; “GenBank: MF988353” for body louse LNSP2; “GenBank: MF997433” for head louse LNSP1; “GenBank: MF997434” for head louse LNSP2).
