# Supplementary material for: Characterization of the human head louse nit sheath reveals proteins with adhesive property that show no resemblance to known proteins
Source: Sci Rep. 2019 Jan 10;9:48. doi: 10.1038/s41598-018-36913-z (PMC6328571; doi:10.1038/s41598-018-36913-z)
Supplement: Supplementary file 1 — SI Results, SI Methods, and SI Figures [file 41598_2018_36913_MOESM1_ESM.pdf]

Supplementary Information for

Characterization of the human head louse nit sheath reveals proteins with adhesive property that show no resemblance to known proteins.

(Running title: Characterization of proteins in the human louse nit sheath)

Jeong Kuk Park[1,‡], Yu Jin Han[1,‡], Jae Ho Lee[2], Sang-Woo Joo[3], Ju Hyeon Kim[4], Si Hyeock Lee[2,4*], and SangYoun Park[1*]

[1]School of Systems Biomedical Science, Soongsil University, Seoul 06978, Republic of Korea

[2]Entomology Division, Department of Agricultural Biotechnology, College of Agriculture and Life Science, Seoul National University, Seoul 08826, Republic of Korea

[3]Department of Chemistry, Soongsil University, Seoul 06978, Republic of Korea

[4]Research Institute of Agriculture and Life Sciences, Seoul National University, Seoul 08826, Republic of Korea

[‡]These authors contributed equally.

[*]Corresponding authors: shlee22@snu.ac.kr, psy@ssu.ac.kr

SI Results

Comparisons of LNSP1 and LNSP2 sequences in head and body lice

The deduced amino acid sequences of LNSP1 and LNSP2 from the head louse of the South Florida strain (HL_SoFl) were compared to that of the body lice BL_Culp and BL_SaFr (Fig. S6). Of note, the overall amino acid sequences of HL_SoFl LNSP1 and LNSP2 were deduced from the new Sanger sequences of this study. Interestingly, many differences in the amino acid sequences of LNSP1 and LNSP2 were observed between head and body lice (Fig. S6). Some of the head louse sequences were either identical to that of the BL_Culp sequence or to the BL_SaFr sequence determined in this study, but some were not conserved in either of the sequences from the body lice. The differences found between the body and head LNSP1/2 occurred in all the three domains of the protein.

For example in LNSP1, slight differences occurred in the signal sequence and in the following regions of the N-terminal polyGA domain where 4-residue and 15-residue insertions took place only in the HL_SoFl LNSP1 (Fig. S6A). Other than the several single residue changes throughout the proteins, differences were especially evident in the C-terminal polyQ domain. The numbers of glutamine residues differed in 1–4 residues throughout all the polyQ regions, and only HL_SoFl showed a polyQ9 region which was commonly found in LNSP2 (Fig. S6A). Overall, the amino acid differences for head louse LNSP1 were 4.0% (compared to the BL_SaFr LNSP1 sequence determined in this study) and 2.6% (compared to the BL_Culp LNSP1 sequence).

Notable differences in LNSP2 between head and body lice other than the single residue changes throughout the proteins included a 21-residue deletion in the N-terminal polyQA domain in HL_SoFl LNSP2 as well as differences in the numbers of glutamine residues in the C-terminal polyQ domain with up to four residue changes in the polyQ4 region. Overall, the amino acid differences between head and body lice LNSP2 were 3.9% (HL_SoFl *vs*. BL_SaFr) and 3.3% (HL_SoFl *vs*. BL_Culp). As expected, sequence variations between head and body lice LNSP1s (and LNSP2s) were higher than the 1.6% amino acid differences (*see below*) for the polymorphisms between LNSP1s (and LNSP2s) in the BL_Culp and BL_SaFr sequences.

Comparisons of polymorphisms in body louse LNSP1 and LNSP2

Some single residue variants as well as deletion and insertion changes were identified in human body louse LNSP1 and LNSP2 when our sequence from the body louse San Francisco strain (BL_SaFr) and the previously reported 2010 sequence of the body louse Culpepper strain (BL_Culp) [12] (noted as the BL_Culp sequence hereafter) were compared. Of note, both sequences are results obtained using Sanger-sequencing. When the sequences of BL_Culp and BL_SaFr were compared, LNSP1 variants with single-residue change; Ala12Pro, Ala13Pro, Val16Leu, and Gly73Arg, were observed as well as Val419 to Ala423 (note the residue number change due to an insertion, *see below*) (Fig. S6A). Also, three residues in the BL_Culp sequence, Ala217-Ala218-Ala219, were changed to Gly217-Gly218-Val219-Gly220-Ala221-Gly222 in the BL_SaFr sequence of LNSP1. Lastly, there were three notable changes in the polyQ regions of LNSP1. First, the nine consecutive Gln in the BL_Culp sequence was increased to ten consecutive Gln in the BL_SaFr sequence by an insertion of a Gln between Gln322 and Gln323 in the BL_Culp sequence. Second, a deletion of Gln336 in the ten consecutive Gln in the BL_Culp sequence resulted in a shortened nine consecutive Gln in the BL_SaFr sequence. Third, an addition of a Gln between Gln379 and Gln380 in the six consecutive Gln in the BL_Culp sequence resulted in a seven consecutive Gln in the BL_SaFr sequence. Overall, 1.6% of the LNSP1 amino acids were different between the body lice BL_Culp and BL_SaFr sequences.

The sequence differences in LNSP2 when the two Sanger sequences above were similarly compared included single residue change variants of His150, Asp329, and Ala360 in the BL_Culp sequence to Gln144, Ala327, and Ser362 in the BL_SaFr sequence (note the residue number changes both from a further deletion and insertion, *see below*) (Fig. S6B). Furthermore in the BL_SaFr sequence, six residues in the BL_Culp sequence, Gln120-Val121-Gln122-Ala123-Gln124-Ala125, were deleted, whereas Ala-Gln-Ala were inserted in the BL_SaFr sequence between Ala174 and Gln175 of the BL_Culp sequence. Also, residues of Ala-Glu-Ala-Lys were inserted in the BL_SaFr sequence between Glu330 and Ala331 of the BL_Culp sequence. Unlike LNSP1, no polymorphisms were observed in the C-terminal polyQ region of LNSP2. Overall, 1.6% of the LNSP2 amino acids were different between the body lice BL_Culp and BL_SaFr sequences.

SI Materials and Methods

Human head louse nit sheath separation

According to the Korean bioethics and safety act, this research was exempt from ethical approval and the subjects or legal guardians provided informed consents. Human head lice eggs and nits (empty egg cases) were collected from five individuals in a family infested with head lice. Because the individuals are of one family, all eggs and nits were considered genetically related. The eggs and nits were manually separated from the hair shaft and placed into a Petri dish with a total of ~200 eggs/nits collected. The obtained eggs and nits were wetted with a few drops of water to minimize static electricity during handling, and moved into a 1.5 mL micro-tube using a small spatula. The louse embryos inside the egg cases were chemically dissolved and eliminated from the egg case (leaving a nit) using the following method. First, the egg-water mixture was centrifuged (5,000×*g* for 1 min) and the water supernatant removed by pipetting. The remaining eggs were treated with 25 μL of buffer containing 12 M urea, 74 mM Tris base, and 78 mM dithiothreitol (DTT), which is a buffer commonly used to dissolve animal keratin [20]. The mix was incubated at 25 °C for 24 hrs. During the incubation, the transparent mixture turned to a clear pale-brownish color. When observed under the microscope, no embryos were seen in the egg cases, thus transforming them into 100% nits. The gross morphology of the nits under the microscope appeared identical to that of the intact eggs except for the disappearance of the embryo. To remove any other contaminating proteins, nits were further treated with a 100 μL of 4% sodium dodecyl sulfate (SDS) and 5 mM DTT at 60 °C for 24 hrs. The morphology of the nits before and after this treatment was also identical, and the nit sheath remained undissolved. The obtained nits were then washed five times with 1 mL aliquots of water by repeated centrifugation and removal of the supernatant as above. The obtained nit sheath failed to dissolve in organic solvents (such as 100% DMSO or cyclohexane) nor in detergent such as 0.01% Triton™ X-100.

Mass spectrometry analysis of the dissolved embryo

The pale-brownish supernatant of the dissolved embryo was analyzed to identify its protein content. First, 5 μL of 0.1 M DTT was added to a 25 μL aliquot of the supernatant, and incubated at 25 °C for 10 mins. For SDS-PAGE analysis, 10 μL of this mixture was mixed with

10 μL of a commercial SDS-PAGE sample buffer (Invitrogen, Carlsbad, CA, USA), and boiled for 10 mins. The mixture was gel-electrophoresed on SDS-PAGE with 12% polyacrylamide (resolving) and 5% (stacking) gel using a running buffer of 25 mM Tris, 0.1% SDS, and 200 mM glycine (100V, 60 mins) and their tryptic digestion fragments were analyzed with LC-MS/MS at a mass spectrometry facility in National Instrumentation Center for Environmental Management (NICEM) (College of Agriculture and Life Sciences, Seoul National University, Seoul, Korea).

Infrared (IR) micro-spectroscopy measurements

An IR micro-spectrum on a single head louse nit that is devoid of the embryo was obtained using a Vertex-80V/Hyperion2000 vacuum FTIR microscope spectrometer (Bruker, Billerica, MA, USA). The vacuum condition was maintained in both the sample compartment and the optics chamber. Deuterated L-alanine doped triglycine sulfate (DLATGS) detector was used to obtain the IR spectrum, and the nominal resolution was 4 cm^{-1} with 32 scans in the spectral range of 4000–650 cm^{-1}. The deconvolution of the amide I bands were further performed using a method in a Peakfit program (Systat, San Jose, CA, USA).

Amino acid composition analysis of the nit sheath

Five nits prepared as above were used for the amino acid composition analysis at the Korea Basic Science Institute (KBSI, Seoul, Korea). The samples were completely air dried over 5 days to remove water, and then hydrolyzed using 50 μL of 6 N HCl at 110 °C for 24 hrs. The hydrolyzed amino acids were modified with phenylisothiocyanate (PITC) using 20 μL of PITC-solution [methanol:H_2O:triethylamine(TEA):PITC=7:1:1:1] at RT for 30 mins. After removing the solvents using a vacuum rotary evaporator, the remaining PITC-modified amino acids were re-dissolved in 200 μL of 140 mM sodium acetate (pH 6.1), 0.15% TEA, 0.03% EDTA, and 6% CH_3CN in water, followed by filtration using a 0.45 μm filter (Millipore, Burlington, MA, USA). After further centrifugation, the supernatant was removed and loaded onto the auto-sampler of HPLC (Hewlett Packard 1100) (Agilent, Santa Clara, CA, USA) for injection into a column (Nova-Pak C18 4 μm, 3.9×300 mm) (Waters, Milford, MA, USA). The elution was performed using a linear gradient of 60% CH_3CN and 0.015% EDTA for 30 mins (1.0 mL/min flow rate). The PITC-derivatized amino acid standards were run using the same condition. Only 18 amino

acids excluding cysteine and tryptophan were expected due to the harsh HCl hydrolysis condition that degrades cysteine and tryptophan. The individual areas under the peaks of the sample chromatogram were compared to that of the amino acid standards to identify and determine the amino acid type and their relative mole percent (Table 1).

Bioinformatic search for candidate nit sheath protein

The complete protein list obtained from the whole genome sequencing of *P. h. corporis* (human body louse) was downloaded from NCBI [12] in order to search for the louse protein whose amino acid composition corresponds to the amino acid analysis result of the nit sheath. The amino acid compositions of 10,773 proteins were calculated using the Biostrings package in a statistical computing program R [37]. A root sum squares (R) in the composition offsets of the 18 amino acids (excluding cysteine and tryptophan) between each of the 10,773 proteins and the experimentally determined louse nit sheath were calculated. The calculated R values ranged from 0.083 to 0.506, and the list of four proteins with the lowest R is shown in Table S2. For instance, the protein with the lowest R of 0.083 was a hypothetical gene of unknown function Phum_PHUM596000 (NCBI Reference Sequence: XP_002432622.1) which encodes 569 amino acids.

PCR amplification of candidate nit sheath protein genes

To confirm that the genes of the identified candidate nit sheath proteins are transcribed in lice, PCR amplifications of the genes were performed using body louse cDNA. Body louse cDNA was generated from 10 females of San Francisco human body louse strain (originated from a homeless man in San Francisco, CA, USA), noted as HL_SaFr, using a Superscript IV Reverse Transcriptase (Invitrogen, Carlsbad, CA) (*see below*), and 10 ng was used for a single 50-µL PCR reaction. The PCR product was electrophoresed at 100 V power for 40 min on an ethidium bromide (0.5 µg/mL) containing 2% agarose gel that was made using the running buffer of 110 mM Tris pH 8.3, 90 mM borate and 2.5 mM EDTA. For complete full-length sequencing, PCR primers were designed to contain the sequences of the 5’ start and the 3’ stop codons of a given gene using the information from the reference genome shotgun sequence (Table S3). The PCR products were fully sequenced using the Sanger method (Bioneer, Daejon, Korea) with the primers used for PCR amplification (Table S3).

Head louse sample collection

The South Florida human head louse strain (originally collected in South Florida, USA, noted as HL_SoFl) was maintained as previously described [38], and various developmental stages of lice (neonate; 5-day nymph; 1- and 5-day old males; 1- and 5-day old females) were collected for total RNA extractions. The 5-day old females were at the active egg-laying stage. All collected lice were flash frozen with liquid nitrogen and stored at -80 °C until use.

Total RNA extraction and cDNA synthesis

Frozen lice (20~30 neonates, 10 5-day old nymphs, 8 males and 8 females per replicate) were homogenized in TRI Reagent (MRC Inc., Cincinnati, OH, USA) by using disposable plastic pestles and tubes. Aliquots of 70 μL or 150 μL TRI reagents were used for the homogenization of neonates or other developmental stage samples, respectively. The homogenates were further processed according to the manufacturer’s instruction. Extracted RNA was treated with DNaseI (Takara, Shiga, Japan) for 25 mins at 37 °C. The cDNA was synthesized from 1 μg total RNA with Superscript IV Reverse Transcriptase (Invitrogen, Carlsbad, CA, USA) using 50 μM oligo d(T)20 for 10 mins at 52.5 °C. The resulting cDNA (approximately 1 μg) was diluted 5-fold with water and used as templates for PCR.

As for the female organ-specific RNA extraction, following dissection of 5-day old females in phosphate-buffered saline under a stereo microscope, the accessory gland, ovary and alimentary tract were isolated and immediately transferred to the RNAlater solution (100 μL) (Ambion, Austin, TX, USA) until sufficient number of organs were obtained (15~20 organs each). The procedures for total RNA extraction and cDNA synthesis were the same as described above.

Quantifications of LNSP1 and LNSP2 transcripts by real-time PCR

To determine the temporal transcription profiles of LNSPs, quantitative real-time PCR (qPCR) was conducted using the head louse cDNA obtained from whole bodies of differential developmental stages (neonate, 5-day old nymph, 1-day old male, 5-day old male, 1-day old

female and 5-day old female) as templates. To determine the spatial transcription profiles of LNSPs, qPCR was conducted using the cDNA obtained from different organs (accessory gland, ovary and alimentary tract) of 5-day old gravid females. qPCR was conducted in 10 µL reactions containing 5 µL SYBR Premix Ex. Taq II (Takara, Shiga, Japan) and 5 pmole of sequence specific primers (Table S4) using Light Cycler 96 (Roche Diagnostics, Basel, Swiss) following the manufacturer's protocol. The following thermal program was used for qPCR: pre-incubation at 95 °C for 30 s, followed by 45 cycles of 95 °C for 5 s, 57 °C for 20 s and 72 °C for 15 s. Relative LNSP transcription levels were estimated using the 2-ΔΔCq formula [39] with actin-5c (for the temporal transcription profiling) or RpL13A (for the spatial transcription profiling) as the internal reference gene. The transcription level of actin-5c was not significantly different (p = 0.2906~0.999, ANOVA in conjunction with Tukey's test) either between different developmental stages or between sexes as judged by Cq values. Similarly, the transcription level of RpL13A was not significantly different (p = 0.4653~0.8773, ANOVA in conjunction with Tukey's test) between different female organs. The PCR efficiencies for all three genes (i.e., LNSP1, LNSP2, actin-5c and RpL13A) were 99.3~106% when using the serially diluted standard templates (cDNA of the whole body or accessory gland of 5 day-old female). Following qPCR, authenticity of PCR product was verified both by melting point analysis and agarose gel electrophoresis. The authentic melting points for LNSP1 and LNSP2 were 83.8 °C and 84.8 °C, respectively, whereas the correct sizes of LNSP1 and LNSP2 amplicons were 89 bp and 128 bp, respectively. Only when LNSP-specific amplification was confirmed, the acquired Cq values were processed for quantification. When non-specific amplification was detected, the machine-reported Cq values were discarded, and the Cq values for the LNSP were regarded as infinite. All qPCR reactions were conducted in three replicates to determine the errors in the experiments.

Partial body louse LNSP1 protein expression and purification

The DNA encoding only the N-terminal and middle domains of LNSP1 (without the putative signal sequence) in human body louse (19–303 of full-length 1–438) were PCR amplified with primers (Table S5) having *Nde*I and *EcoR*I restriction enzyme sites and body louse cDNA as the template. The PCR product was cloned into pET28a vector (Merck, Kenilworth, NJ, USA) for expression in *E. coli* and affinity purification with an N-terminal His_6-

tag. The plasmid generated was sequence verified (Bioneer, Daejon, Korea) of the insert region, and were used to transform *E. coli* BL21 (DE3) (Merck, Kenilworth, NJ, USA) cells using heat shock at 42 °C (45 sec). The transformed cells were grown at 37 °C in 1 L of Luria-Bertani (LB) medium to an OD_{600} of ~0.8 in the presence of 25 μg/mL kanamycin. Expression of the recombinant partial LNSP1was induced by addition of 0.5 mM isopropyl-D-thiogalactopyranoside (IPTG) at 22 °C, and cells were allowed to grow for extra 16 h. Cells were harvested using centrifugation at 4500×*g* for 10 min (4 °C). The partial LNSP1 with N-terminal His_6-tag was over-produced with soluble expression of the protein. For protein purification, the bacterial cell pellets were re-suspended in 50 mL ice-cold lysis buffer (20 mM Tris pH 7.5, 500 mM NaCl, and 5 mM imidazole) and lyzed on ice by sonication. The homogenates were centrifuged at 70000×g for 30 min (4 °C), and supernatants poured over 5 mL Ni-nitrilotriacetic acid agarose (Ni-NTA) (Qiagen, Hilden, Germany) gravity column. The columns were washed with five column volumes of wash buffer (20 mM Tris pH 7.5, 20 mM imidazole, and 500 mM NaCl), and the proteins were eluted with elution buffer (20 mM Tris pH 7.5, 200 mM imidazole, and 500 mM NaCl). The elution fractions containing the partial LNSP1 were checked using Bradford assay (BioRad, Berkeley, CA, USA), combined, and added with 50 μL of 0.25 U/μL bovine thrombin (Invitrogen, Carlsbad, CA, USA). After proteolysis of the His_6-tag for 16 h incubation at 4 °C, the protein samples were further purified using HiLoad® 26/60 Superdex® 200 size-exclusion column (SEC) pre-equilibrated with SEC buffer (50 mM Tris pH 7.5, 150 mM NaCl, and 2 mM DTT). The proteolysis mixture was loaded into the column connected to an ÄKTA FPLC system (GE Healthcare, Little Chalfont, UK). The elution profile showed one major peak, and the fractions were concentrated by Amicon® ultracentrifugation filtration system (Merck, Kenilworth, NJ, USA).

Adhesive strength measurements

Adhesive strength of expressed partial body louse LNSP1 on the surface of polypropylene (PP) film was measured using a universal testing machine (UTM, Instron model 5543, Norwood, MA, USA). 20 μL of the expressed partial LNSP1 which was concentrated to ~2 mg/mL (40 μg) was applied onto a PP film over an area of 2 cm × 5 cm, overlaid with another PP film, and allowed to completely dry for 1 day before testing. For controls, chymotrypsin (Sigma Aldrich, St. Louis,

MO, USA) and bovine serum albumin (Bioshop, Burlington, ON, Canada) were applied on to the PP films using the same concentration (2 mg/mL) and amount (40 μg) as in the case of partial LNSP1. As a positive control, a commercially available fibrin-based sealant (Tisseel®, Baxter, Deerfield, IL, USA) was used to glue together two PP films similarly. Fibrin and thrombin separately inside two syringes were mixed to glue together two PP films using a final volume of ~200 μL fibrin solution. ~18 mg of fibrin was calculated to cover the area of 2 cm × 5 cm when reasoned from the manufacturer's protocol which indicates ~90 mg of fibrin in 1 mL of fibrin-containing syringe. Usage of ~500-fold more of fibrin (in grams) in comparison to LNSP1 was unavoidable because of the viscous nature of the fibrin-thrombin mix. Fibrin-glued PP films were allowed to dry for only 1 day as well. Tensile forces were applied on the glued PP films at a rate of 10 mm/min, and the force-distance curve measured until the films completely broke apart. The forces needed to break the PP films were averaged for at least three samples in each case for comparison of their adhesive properties.

References

12. Kirkness, E. F., Haas, B. J., Sun, W., Braig, H. R., Perotti, M. A., Clark, J. M. *et al.* Genome sequences of the human body louse and its primary endosymbiont provide insights into the permanent parasitic lifestyle. *Proc Natl Acad Sci U S A.* **107(27)**, 12168–12173 (2010).

14. Kang, J. S., Cho, Y -J., Kim, J. H., Kim, S. H., Yoo, S. Noh, S- J., Park, J., Yoon, K. S., Clark, J. M., Pittendrigh, B. R., Chun, J. & Lee S. H. Comparison of the genome profiles between head and body lice. *J. Asia-Pacific Entomol.* **18(3)**, 377–382 (2015).

20. Sahajpal, V., Goyal, S., Singh, K. & Thakur, V. Dealing wildlife offences in India: role of the hair as physical evidence. *Int J Trichology* **1(1)**, 18–26 (2009).

37. Pagès, H., Aboyoun, P., Gentleman, R. & DebRoy, S. *Biostrings: String objects representing biological sequences, and matching algorithms*. R package version 2.42.1 (2017).

38. Kim, J. H., Min, J. S., Kang, J. S., Kwon, D. H., Yoon, K. S., Strycharz, J., Koh, Y. H., Pittendrigh, B. R., Clark, J. M. & Lee, S. H. Comparison of the humoral and cellular immune

responses between body and head lice following bacterial challenge. *Insect Biochem Mol Biol.* **41(5)**, 332–339 (2011).

39. Livak, K. J. & Schmittgen, T. D. Analysis of relative gene expression data using real-time quantitative PCR and the $2^{-\Delta\Delta Ct}$ method. *Methods.* **25(4)**, 402–408 (2001).

Supplementary Figure Legends

SFigure 1. The nits treated with the embryo dissolving buffer. The eggs of human head louse were observed under a microscope before and after the treatment with 12 M urea, 74 mM Tris base, and 78 mM dithiothreitol (DTT). The nits devoid of embryos (*right*) were used for the amino acid composition analysis and FTIR analysis. The pale-brownish supernatant of the dissolved embryo was analyzed using SDS-PAGE and was subjected for mass spectrometry analysis. The results identified many proteins of louse origin (Table S1).

SFigure 2. Amino acid elution chromatogram of HCl-hydrolyzed nit sheath samples. (A) Amino acid composition analysis was performed on the PITC-derivatives of 20 amino acid standards (in *red*) and further on the nit sheath samples. (B) The sample chromatogram was compared with the standards chromatogram (in *red*) to determine the amino acid types and the relative mole percentage of the amino acids composing the nit sheath.

SFigure 3. PCR amplification of PHUM595880 and PHUM596000 using body lice cDNA. (A) PCR amplifications of the four candidate louse nit sheath genes were performed by using the body louse cDNA which was generated from female lice (San Francisco strain) mRNA. Among the four candidate genes, the DNAs of PHUM595880 (~1300 bp, Lane 1) and PHUM596000 (~1700 bp, Lane 2) were obtained (M, Marker).

SFigure 4. Amino acid sequences of PHUM403440 (A, B) and PHUM595890 (C). (A) The amino acid sequence of the partially determined body louse PHUM403440 (2010 sequence, ref.12) using the Culpepper body louse (BL_Culp) strain is identical to our sequenced body louse PHUM595880 using the San Francisco (BL_SaFr) strain (*this study*). (B) The head louse version of body louse PHUM403440 was searched in the whole genome of head louse sequenced using the Bristol head louse (HL_Bris) strain (2015 sequence, ref.14) and a homolog with 98.7% match was found. The HL_Bris PHUM403440 is 95.8% identical to our sequenced PHUM595880 using the South Florida head louse (HL_SoFl) strain (*this study*). (C) The protein product of BL_Culp PHUM595890 with a total of 127 amino acids (Table S2) is different from our BL_SaFr PHUM596000 (*this study*) mostly in the N-terminal 37 residues holding the

signal sequence, and is nearly identical to BL_SaFr PHUM596000 with only two different residues out of the remaining 90 residues in C-terminal region.

SFigure 5. Domain structures of LNSP1 and LNSP2 in head and body lice. The amino acid sequences of LNSP1 and LNSP2 in head louse (A and B), and in body louse (C and D) are shown with indications of signal sequence (in *blue italic*), the N-terminal polyQA domain, the middle polyGA domain, and the C-terminal polyQ domain. Conserved residues of Gln (capitalized in *red*) and Gly (in *yellow* background) are shown. The protein sequences are translated from our DNA sequencing results of South Florida head louse (HL_SoFl) strain and San Francisco body louse (BL_SaFr) strain (*this study*).

SFigure 6. Polymorphisms observed in LNSP1 (A) and LNSP2 (B). (A) LNSP1 regions with amino acid differences are indicated (with *bold* type) in our South Florida head louse (HL_SoFl) strain sequence (*this study*), our San Francisco body louse (BL_SaFr) strain sequence (*this study*), and the 2010 Culpepper body louse (BL_Culp) strain sequence [12]. Regions that are different between two body louse strains (BL_SaFr and BL_Culp) are also highlighted (in *yellow*). (B) LNSP2 regions with amino acid differences are indicated (with *bold* type) in our South Florida head louse (HL_SoFl) strain sequence (*this study*), our San Francisco body louse (BL_SaFr) strain sequence (*this study*), and the 2010 Culpepper body louse (BL_Culp) strain sequence [12]. Regions that are different between two body louse strains (BL_SaFr and BL_Culp) are also highlighted (in *yellow*).

Table S1. List of proteins identified using MS analysis on the supernatant of urea-treated head louse eggs (only proteins with >10% coverage of sequence is listed)

Accession	Description	Coverage
XP_002431396.1	Vitellogenin-1 precursor, putative [Pediculus humanus corporis]	54.4
XP_002424815.1	Vitellogenin-1 precursor, putative [Pediculus humanus corporis]	42.8
XP_002430692.1	protein lethal, putative [Pediculus humanus corporis]	39.5
XP_002424678.1	hemocyanin subunit, putative [Pediculus humanus corporis]	38.6
XP_002429121.1	protein lethal, putative [Pediculus humanus corporis]	35.1
XP_002426240.1	apolipophorin-III, putative [Pediculus humanus corporis]	30.2
XP_002430576.1	Endocuticle structural glycoprotein SgAbd-2, putative [Pediculus humanus corporis]	29.2
XP_002424677.1	tubulin alpha-1 chain [Pediculus humanus corporis]	28.4
XP_002423709.1	Actin-5C [Pediculus humanus corporis]	25.8
XP_002423626.1	ATP synthase subunit beta, putative [Pediculus humanus corporis]	25.5
XP_002424669.1	apolipophorins precursor, putative [Pediculus humanus corporis]	24.6
XP_002424793.1	elongation factor-1 alpha, partial [Pediculus humanus capitis]	21.7
XP_002428823.1	elongation factor-1 alpha, partial [Pediculus humanus capitis]	21.7
XP_002430633.1	tubulin beta-1 chain [Pediculus humanus corporis]	21.7
XP_002431396.1	Actin, muscle [Pediculus humanus corporis]	20.7
XP_002431398.1	hypothetical protein Phum_PHUM146140 [Pediculus humanus corporis]	20.2
XP_002424678.1	Actin-5C [Pediculus humanus corporis]	19.6
XP_002424685.1	Actin, muscle [Pediculus humanus corporis]	19.4
XP_002423709.1	alpha/beta-gliadin A-I precursor, putative [Pediculus humanus corporis]	18.9
XP_002424670.1	conserved hypothetical protein [Pediculus humanus corporis]	18.4
XP_002424677.1	cutile protein, putative [Pediculus humanus corporis]	17.2
XP_002430692.1	Heat shock 70 kDa protein cognate, putative [Pediculus humanus corporis]	16.5
XP_002429368.1	hemocyanin subunit, putative [Pediculus humanus corporis]	15.4
XP_002426688.1	conserved hypothetical protein [Pediculus humanus corporis]	14.8
XP_002424669.1	conserved hypothetical protein, partial [Pediculus humanus corporis]	14.8
XP_002422844.1	hypothetical protein Phum_PHUM146230 [Pediculus humanus corporis]	13.8
XP_002432207.1	Heat shock 70 kDa protein cognate, putative [Pediculus humanus corporis]	13.3
XP_002431066.1	ATP synthase subunit beta, putative [Pediculus humanus corporis]	13.3
XP_002432822.1	cutile protein, putative [Pediculus humanus corporis]	12.9
XP_002424686.1	elongation factor 1-alpha, putative [Pediculus humanus corporis]	12.4
XP_002430633.1	40S ribosomal protein S9, putative [Pediculus humanus corporis]	10.8
XP_002430661.1	Heat shock 70 kDa protein cognate 3 precursor, putative [Pediculus humanus corporis]	10.1

Table S2. Low R-valued candidates for the body louse nit sheath protein shown with calculated amino acid contents*

Protein	R	Ala (%)	Glx (%)	Gly (%)	Val (%)	Protein ID (Locus)	No. of amino acids	Predicted MW (kDa)
Experimental amino acid composition of nit	-	20.2	24.4	25.4	10.0	-	-	-
PHUM596000	0.083	21.8	23.8	19.7	11.3	XP_002432622.1	569	55.0
PHUM595880	0.095	17.0	20.7	20.7	13.3	XP_002432620.1	434	43.1
PHUM403440	0.138	14.2	30.2	18.3	11.8	XP_002428823.1	>169	>17.3
PHUM595890	0.139	14.2	17.3	18.9	9.4	XP_002432621.1	127	12.8

*The amino acid contents of Ala, Glx, Gly, and Val are calculated excluding the Cys and Trp residues in the individual protein sequences.

Table S3. Primers for PCR of PHUM595880 (LNSP1) and PHUM596000 (LNSP2) in body and head lice

Primer name	Primer sequence (5′-3′)	Remark
Body Louse_ PHUM595880_F	GTGTACTTGTGAGCTACTA	For the amplification of LNSP1
Body Louse_ PHUM595880_R	TTAGTAAACTAGGCCACGGGC	
Body Louse_ PHUM596000_F	TGCAAATATTGTAAAATTCAAAA	For the amplification of LNSP2
Body Louse_ PHUM596000_R	TTAGTAGACGACGGAACCAGA	
Head Louse_ PHUM595880_F	TGTGTACTTGTGAGCTACTAC	For the amplification of LNSP1
Head Louse_ PHUM595880_F	CTAATTAGTCGTTTAGTAAACTAGG	
Head Louse_ PHUM596000_F	TAATCCAAATGTACTTCAAAACTTC	For the amplification of LNSP2
Head Louse_ PHUM596000_F	TGAGTATGAACCTAGTAGACG	

Table S4. Primers for quantitative real-time PCR (qPCR) on head louse cDNA

Primer name	Primer sequence (5′-3′)	Remark
Head Louse_LNSP1_F	GAGTCGCTCGCTCTGAATC	For the amplification of LNSP1
Head Louse_LNSP1_R	GGAAACGTGAGCTACGGTG	
Head Louse_LNSP2_F	AATGGGTGGAATGAGCCAATC	For the amplification of LNSP2
Head Louse_LNSP2_R	CGACAGAGGGGTAGAAAGC	
Head Louse_Actin-5C_F	GTTTTATTGACCGAAGCTCCTC	For the amplification of actin-5C, an internal reference gene
Head Louse_Actin-5C_R	CAAGACAGCTTGAATAGCAACG	

Table S5. Primers for recombinant partial LNSP1 (19-303) expression in pET28a

Primer name	Primer sequence (5′-3′)	Remark
Body Louse_LNSP1_F19	ggcCATATGGGTGTCTGGCATGG	For the amplification of LNSP1 from 19-303 (*Nde*I/*EcoR*I)
Body Louse_LNSP1_R303	ttcGAATTCTTAAGCGACGGTAGCAACGT	

Supplementary Figures

Figure S1

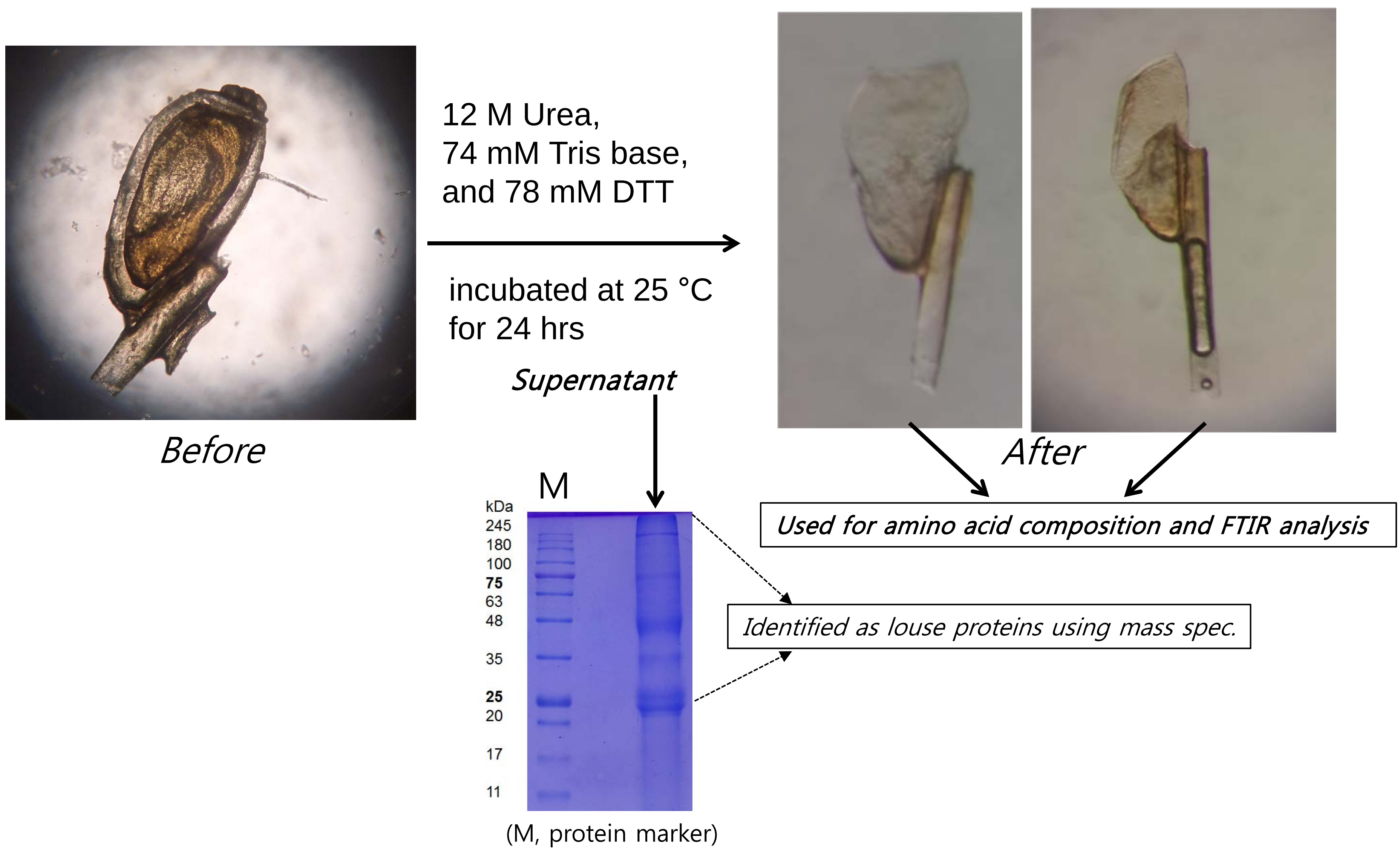
12 M Urea,
74 mM Tris base,
and 78 mM DTT
incubated at 25 °C
for 24 hrs
Supernatant
Before
After
M
kDa
245
180
100
75
63
48
35
25
20
17
11
(M, protein marker)
Used for amino acid composition and FTIR analysis
Identified as louse proteins using mass spec.

Figure S2A

A

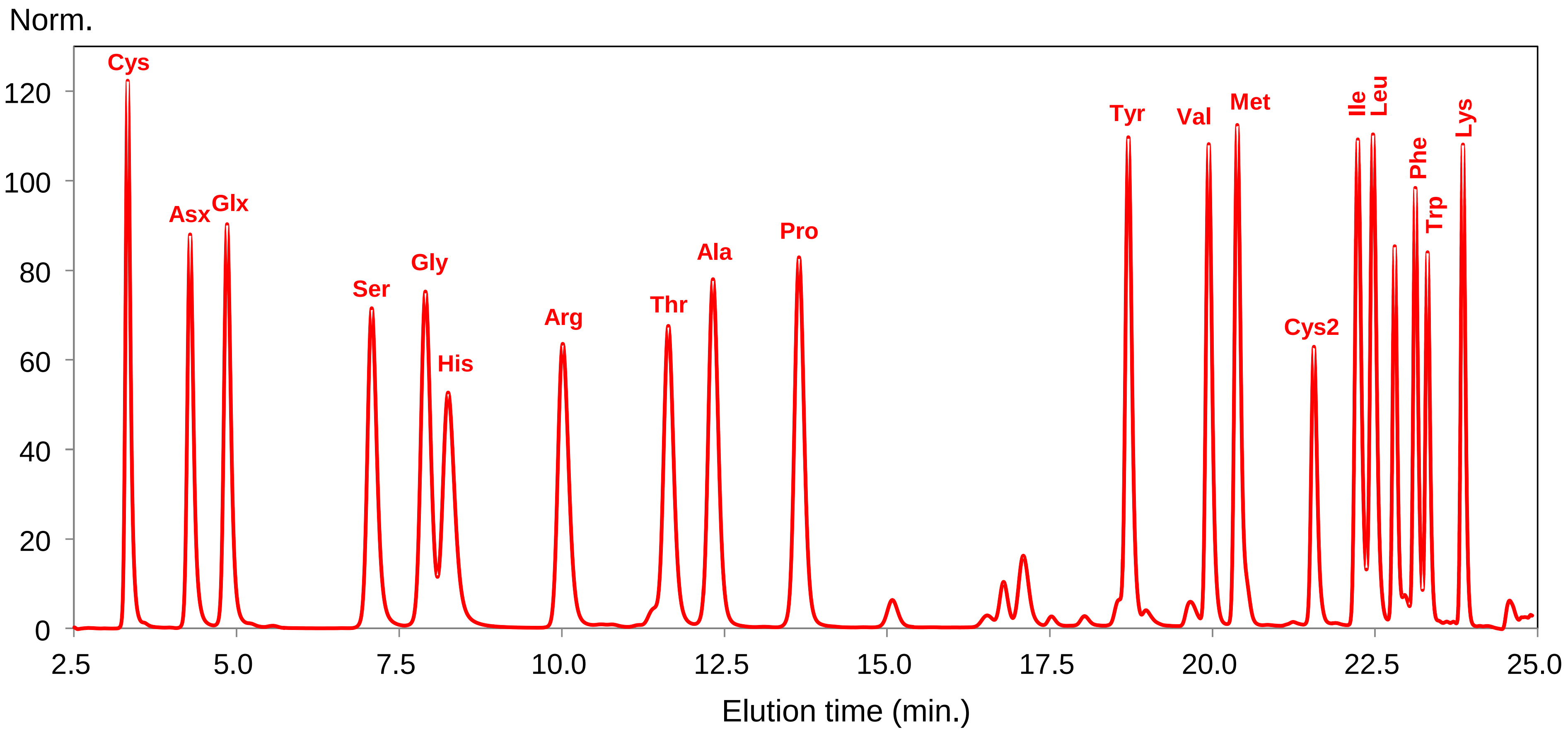

Norm.
120
100
80
60
40
20
0
Cys
Asx
Glx
Ser
Gly
His
Arg
Thr
Ala
Pro
Tyr
Val
Met
Cys2
Ile
Leu
Phe
Trp
Lys
2.5
5.0
7.5
10.0
12.5
15.0
17.5
20.0
22.5
25.0
Elution time (min.)

Figure S2B

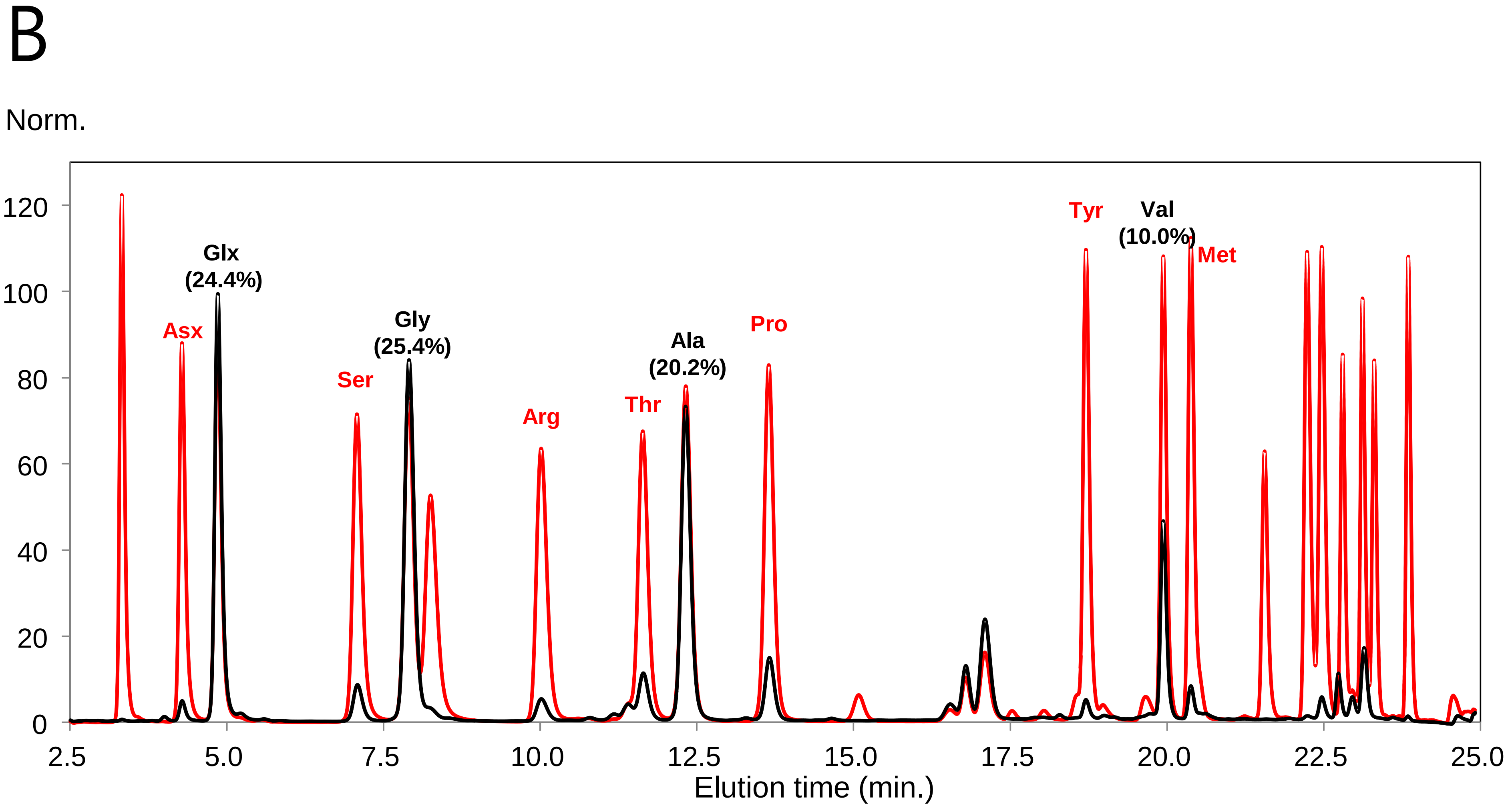

B
Norm.
0
20
40
60
80
100
120
2.5
5.0
7.5
10.0
12.5
15.0
17.5
20.0
22.5
25.0
Elution time (min.)
Asx
Glx (24.4%)
Ser
Gly (25.4%)
Arg
Thr
Ala (20.2%)
Pro
Tyr
Val (10.0%)
Met

Figure S3

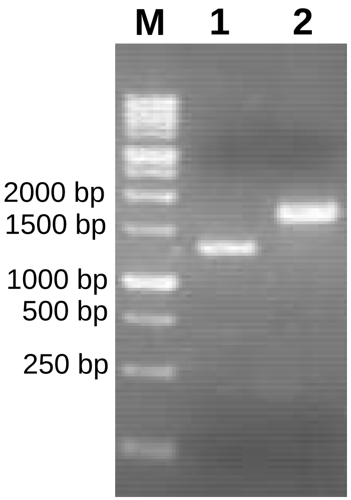

M: DNA Marker
1: LNSP1
2: LNSP2

Figure S4A

```
PHUM403440(BL_Culp)       -(partial sequence)------------------------------------------------
BL LNSP1
PHUM595880(BL_SaFr)    1  mffkfsvfvlfppgflqsgvwhgmwggesQsQshhQsQghQghhmwggyrgvggQaQsQaQaQgQ   65
PHUM595880(BL_Culp)    1  mffkfsvfvlfaagfvqsgvwhgmwggesQsQshhQsQghQghhmwggyrgvggQaQsQaQaQgQ   65

PHUM403440(BL_Culp)       -----------------------------------------------------------------
BL LNSP1
PHUM595880(BL_SaFr)   66  kwggmgmrQaQaQaQgQkwggmgmgQaQaQaQgQsmggmgvaQtQgvvvrgvrtrtvpvvtkves  130
PHUM595880(BL_Culp)   66  kwggmgmgQaQaQaQgQkwggmgmgQaQaQaQgQsmggmgvaQtQgvvvrgvrtrtvpvvtkves  130

PHUM403440(BL_Culp)       -----------------------------------------------------------------
BL LNSP1
PHUM595880(BL_SaFr)  131  gytagfnkgfangftagvarsessqkvvsgfptavymgyptvahvspvvqepvveqvyevvdgpv  195
PHUM595880(BL_Culp)  131  gytagfnkgfangftagvarsessqkvvsgfptavymgyptvahvspvvqepvveqvyevvdgpv  195

PHUM403440(BL_Culp)       -----------------------------------------------------------------
BL LNSP1
PHUM595880(BL_SaFr)  196  vsgvgamagaaaggvgagagaggvgagagaggvgagagaaaaagaggvgggaaaaaaaggveaea  260
PHUM595880(BL_Culp)  196  vsgvgamagaaaggvgagaga---aaaagaggvgagagaaaaagaggvgggaaaaaaaggveaea  257

PHUM403440(BL_Culp)    1  ---------sgaaagvvgtgfqavpatmaylpvmtvphvatvaggQQQQQQQggagvggQQQQQQ   56
BL LNSP1
PHUM595880(BL_SaFr)  258  eteeavvaesgaaagvvgtgfqavpatmaylpvmtvphvatvaggQQQQQQQggagvggQQQQQQ  325
PHUM595880(BL_Culp)  261  eteeavvaesgaaagvvgtgfqavpatmaylpvmtvphvatvaggQQQQQQQggagvggQQQQQQ  322

PHUM403440(BL_Culp)   57  QQQQggagvggQQQQQQQQQ-ggagvggQQQQQQQkQskkvvktvttkvvttkkQkQQQQQQQag  120
BL LNSP1
PHUM595880(BL_SaFr)  325  QQQQggagvggQQQQQQQQQ-ggagvggQQQQQQQkQskkvvktvttkvvttkkQkQQQQQQQag  389
PHUM595880(BL_Culp)  323  -QQQggagvggQQQQQQQQQQggagvggQQQQQQQkQskkvvktvttkvvttkkQkQQQQQQ-ag  385

PHUM403440(BL_Culp)  121  avvggQQQQQQQQhhlagyrtaapvytgyagryagavagaaagarglvy  169
BL LNSP1
PHUM595880(BL_SaFr)  390  avvggQQQQQQQQhhlagyrtaapvytgyagryagavagaaagarglvy  438
PHUM595880(BL_Culp)  386  avvggQQQQQQQQhhlagyrtaapvytgyagryvgavagaaagarglvy  434
```

Figure S4B

```
PHUM403440                 ------------------------------------------------------------------
of HL_Bris
PHUM595880             1   mffkfsvfvlfaagfvqsgvwhgmwggesqsqshhqshhqsqghqghhmwggyrgvggqaqsqa   64
of HL_SoF1(LNSP1)

PHUM403440                 ------------------------------------------------------------------
of HL_Bris
PHUM595880            65   qgqkwggmgmgqaqaqaqgqgwggmgmgqaqaqaqgqgwggmgmgqaqaqaqgqsmggmgvaqt  128
of HL_SoF1(LNSP1)

PHUM403440                 ------------------------------------------------------------------
of HL_Bris
PHUM595880           129   qgvvvrgvrtrtvpvvtkvesgytagfnkgfangftagvarsessqkvvsgfptavymgyptva  192
of HL_SoF1(LNSP1)

PHUM403440                 ------------------------------------------------------------------
of HL_Bris
PHUM595880           200   hvspvvqepvveqvyevvdgpvvsgvgamagaaaggvgagagaaaaagaggvgggagaaaaaga  256
of HL_SoF1(LNSP1)

PHUM403440             1   ---------------------------sgaaagvvgtgfqavpatmaylpvmtvphvatvaggQ   37
of BL_Culp
PHUM403440             1   ---------------------------------------------maylpvmtvpqvatvaggQ   19
of HL_Bris
PHUM595880           257   ggvgggagaaaggveaeaeteeavvaesgaaagvvgtgfhavpasmaylpvmtvphvatvaggQ  320
of HL_SoF1(LNSP1)

PHUM403440            38   QQQQQQggagvggQQQQQQQQQQggagvggQQQQQQQQQ-ggagvggQQQQQQQkQskkvvktvt  101
of BL_Culp
PHUM403440            20   QQQQQQQgagvggQQQQQQQQQeggagvggQQQQQQQQQgggagvggQQQQQQQkQskkvvktvt   83
of HL_Bris
PHUM595880           321   QQQQQQQg-gaggQQQQQQQ---ggagvggQQQQQQQ---ggagvggQQQQQ--kQskkvvktvt  376
of HL_SoF1(LNSP1)

PHUM403440           102   tkvvttkkQkQQQQQQQ-agavvggQQQQQQQQ----------------hhlagyrtaapvytgy  149
of BL_Culp
PHUM403440            84   tkvvttkkQkQQQQQQQ-agavvggQQQQQQQQ----------------hhlagyrtaapvytgy  131
of HL_Bris
PHUM595880           377   tkvvttkkQkQQQQQQQQagavvggQQQQQQQQQQagavvggQQQQQQQhylagyrtaapvytgy  441
of HL_SoF1(LNSP1)

PHUM403440           150   agryagavagaaagarglvy  169
of BL_Culp
PHUM403440           132   agryagavagaaagarglvy  151
of HL_Bris
PHUM595880           442   agryagavagaaagarglvy  461
of HL_SoF1(LNSP1)
```

Figure S4C

```
BL LNSP2
PHUM596000(BL_SaFr)    1 myfktsvlvlvaatfvasypmhgsyggwgsQgQsQsQaQgQmmgghyggmgQgQaQaQsQgQmmgg  66
PHUM595890(BL_Culp)    1 mkrkpivkiitlllspffflmrs-------------------------------------------  24

BL LNSP2
PHUM596000(BL_SaFr)   67 hyggmgQgQaQaQsQgQmmggyyggmgQaQaQaQaQalQaaQaaQvaQaQaQvQaQalkaaQaQ 132
PHUM595890(BL_Culp)      ------------------------------------------------------------------

BL LNSP2
PHUM596000(BL_SaFr)  133 aQaQaQaveaaQaQaQaQavQvaQaQaQavQaaQaQaaQaQaarvvgsrrvvsvmrgmgg 199
PHUM595890(BL_Culp)      ------------------------------------------------------------------

BL LNSP2
PHUM596000(BL_SaFr)  200 msqsftsgfgdgfatgygkgvqtvqgvgsngvvafypsvamafptvasagpsvvqepvveqvyevv 265
PHUM595890(BL_Culp)      ------------------------------------------------------------------

BL LNSP2
PHUM596000(BL_SaFr)  266 dgpvvsgvgamagaaagagfggvgagaaaaagaggvggagaaaaagsgvgagaaaaaaaggveae 330
PHUM595890(BL_Culp)      ------------------------------------------------------------------

BL LNSP2
PHUM596000(BL_SaFr)  331 deaeaeakaeaeaeaevegeetvvaesgaaagaaagasagvvgtgfhavpatmaylpvmtvphvat 396
PHUM595890(BL_Culp)      ------------------------------------------------------------------

BL LNSP2
PHUM596000(BL_SaFr)  397 vaggQQQQQQQggaggQQQQQQQQQggagvggQQQQQeggaggQQQQQQgkagvggQQQQQQkQsk 456
PHUM595890(BL_Culp)      ------------------------------------------------------------------

BL LNSP2
PHUM596000(BL_SaFr)  457 kvvktvttkvvttttkQkQkQkQQagaggQQQQQQQagagvggQQQQQQQeggaggQQQQQQQQgga 522
PHUM595890(BL_Culp)   25 ------------rrptttttrksrtgaggQQQQQQQagagvggQQQQQQQeggaggQQQQQQQ-gga  78

BL LNSP2
PHUM596000(BL_SaFr)  523 ggvvggvvpvaaapvvaapvyhggytggfggaaasaaasavsksgsvvy 571
PHUM595890(BL_Culp)   79 ggvvggvvpvaaapvaaapvyhggytggfggaaasaaasavsksgsvvy 127
```

Figure S5A – HL LNSP1

Signal sequence

*mffkfsvfvlfaagfvqs*gvwhg

polyQA N-terminal domain

mwggesQsQshhQshhQsQghQghh
mwggyrgvggQaQsQaQgQ
kwggmgmgQaQaQaQgQ
gwggmgmgQaQaQaQgQ
gwggmgmgQaQaQgQ
smggmgvaQtQgvvvrgvrtrtvpvvtkvesgytagfnkgfangftagvarsessqkvvsgfptavymgyp
tvahvspvvqepvveqvyevvdgpvvs

polyGA middle domain

-------gvgamaga
aaggvgagag**aaaaa**gag
gvgg-gag--**aaaaa**
gaggvggag**aaa**
ggveaeaeteeavvaesgaaagvvgtgfhavpasmaylpvmtvphvatva

polyQ C-terminal domain

ggQQQQQQQQgga
ggQQQQQQQ-ggagv
ggQQQQQQQ-ggagv
ggQQQQQkQskkvvktvttkvvtt
kkQkQQQQQQQQagavv
ggQQQQQQQQQQagavv
ggQQQQQQQhylagyrt

aapvytgyagryagavagaaagarglv

Figure S5B – HL LNSP2

Signal sequence

*myfktsvlvlvaatfvas*ypmhg

polyQA N-terminal domain

```
syggwgsQgQsQsQaQgQmmgg
hyggmg-QgQaQaQsQgQmmgg
hyggmg-QaQaQaQaQalQaaQaaQva
--------------QaQaQaQvQaQalkaa
--------------QaQaQaQaQaveaa
--------------QaQaQahaQavQva
--------------QaQaQaQaQaaQaQaQaarvvgsrrvvsvmr
```

polyGA middle domain

```
gmggmsqsftsgfgdgfat
gygkgvqtvq
gvgsngvvafypsvamafptvasagpsvvqepvveqvyevvdgpvvs
-----gvgamagaaa
gagcggvgagaaaaa
gaggvgggagaaaa
---gsgvgagaaaaaaa
aaggveaedeaeaeaeaeaeaevegeetvva
esgaaagaaagasagvvgtgfhavpatmaylpvmtvphvatva
```

polyQ C-terminal domain

```
ggQQQQQQQQ--gga
ggQQQQQQQQ--ggarv
ggQQQQQQQe--ggagv
rgQQQQQQQQQQggagv
ggQQQQQQQkQsrkvvktvttkvvtttkQkQkQkQQQQaea
ggQQQQQQ-e--ggaga
ggQQQQQQQe--gga
ggQQQQQQQ---gga
ggvv
ggvv
pvaaa
pvvaa
pvyhggytggfggaaagaaasavsksgsvvy
```

Figure S5C – BL LNSP1

Signal sequence

*mffkfsvfvlfppgflqs*gvwhg

polyQA N-terminal domain

mwggesQsQshhQsQghQghh
mwggyrgvggQaQsQaQaQgQ
kwggmgmrQaQaQaQgQ
kwggmgmgQaQaQaQgQ
smggmgvaQtQgvvvrgvrtrtvpvvtkvesgytagfnkgfangftagvarsessqkvvsgfptavymgyp

tvahvspvvqepvveqvyevvdgpvvs

polyGA middle domain

-------gvgama-ga
aaggvgagagaggvgaga
gaggvgagag**aaaaa**
gaggvggg**aaaaaaa**

ggveaeaeteeavvaesgaaagvvgtgfqavpatmaylpvmtvphvatva

polyQ C-terminal domain

ggQQQQQQQ---ggagv
ggQQQQQQQQQQggagv
ggQQQQQQQQQ-ggagv
ggQQQQQQQkQskkvvktvttkvvtt
kkQkQQQQQQQa-gavv
ggQQQQQQQQhhlagyr

taapvytgyagryagavagaaagarglvy

Figure S5D – BL LNSP2

Signal sequence

*myfktsvlvlvaatfvas*ypmhg

polyQA N-terminal domain

syggwgsQgQsQsQaQgQmmgg
hyggmg-QgQaQaQsQgQmmgg
hyggmg-QgQaQaQsQgQmmgg
yyggmg-QaQaQaQaQalQaaQaaQva
-------------QaQaaQvQaQalkaa
-------------QaQaQaQaQaveaa
-------------QaQaQaQaQavQva
-------------QaQaQaQavQaaQaQaQaaQaQaarvvgsrrvvsvmr

polyGA middle domain

gmggmsqsftsgfgdgfat
gygkgvqtvq
gvgsngvvafypsvamafptvasagpsvvqepvveqvyevvdgpvvs
-----gvgamag**aaa**
gagfggvgag**aaaaa**
gaggvgggag**aaaaa**
---gsgvgag**aaaaa**
aaggveaedeaeaeakaeaeaeaevegeetvva
esgaaagaaagasagvvgtgfhavpatmaylpvmtvphvatva

polyQ C-terminal domain

ggQQQQQQQ--gga
ggQQQQQQQQQggagv
ggQQQQQe---gga
ggQQQQQQgkagv
ggQQQQQQkQskkvvktvttkvvttttkQkQkQkQQaga
ggQQQQQQQagagv
ggQQQQQQQe-gga
ggQQQQQQQQ-gga
ggvv
ggvv
pvaaa
pvvaa
pvyhggytggfggaaasaaasavsksgsvvy

Figure S6A

```
HL_SoFl    1 MFFKFSVFVLFAAGFVQSGVWHGMWGGESQSQSHHQSHHQSQGHQGHHMWGGYRGVGGQAQSQA  64
BL_SaFr    1 MFFKFSVFVLFPPGFLQSGVWHGMWGGESQSQSHH----QSQGHQGHHMWGGYRGVGGQAQSQA  60
BL_Culp    1 MFFKFSVFVLFAAGFVQSGVWHGMWGGESQSQSHH----QSQGHQGHHMWGGYRGVGGQAQSQA  60

HL_SoFl   65 QGQKWGGMGMGQAQAQAQGQGWGGMGMGQAQAQAQGQGWGGMGMGQAQAQAQGQSMGGMGVAQT 128
BL_SaFr   61 ---------------QAQGQKWGGMGMRQAQAQAQGQKWGGMGMGQAQAQAQGQSMGGMGVAQT 109
BL_Culp   61 ---------------QAQGQKWGGMGMGQAQAQAQGQKWGGMGMGQAQAQAQGQSMGGMGVAQT 109

HL_SoFl  129 QGVVVRGVRTRTVPVVTKVESGYTAGFNKGFANGFTAGVARSESSQKVVSGFPTAVYMGYPTVA 192
BL_SaFr  110 QGVVVRGVRTRTVPVVTKVESGYTAGFNKGFANGFTAGVARSESSQKVVSGFPTAVYMGYPTVA 173
BL_Culp  110 QGVVVRGVRTRTVPVVTKVESGYTAGFNKGFANGFTAGVARSESSQKVVSGFPTAVYMGYPTVA 173

HL_SoFl  200 HVSPVVQEPVVEQVYEVVDGPVVSGVGAMAGAAAGGVGAGAGA---AAAAGAGGVGGGAGAAAA 253
BL_SaFr  174 HVSPVVQEPVVEQVYEVVDGPVVSGVGAMAGAAAGGVGAGAGAGGVGAGAGAGGVGAGAGAAAA 237
BL_Culp  174 HVSPVVQEPVVEQVYEVVDGPVVSGVGAMAGAAAGGVGAGAGA---AAAAGAGGVGAGAGAAAA 234

HL_SoFl  254 AGAGGVGGGAGAAA--GGVEAEAETEEAVVAESGAAAGVVGTGFHAVPASMAYLPVMTVPHVAT 315
BL_SaFr  238 AGAGGVGGGAAAAAAAGGVEAEAETEEAVVAESGAAAGVVGTGFQAVPATMAYLPVMTVPHVAT 301
BL_Culp  235 AGAGGVGGGAAAAAAAGGVEAEAETEEAVVAESGAAAGVVGTGFQAVPATMAYLPVMTVPHVAT 298

HL_SoFl  316 VAGGQQQQQQQQGGAGGQQQQQQQ----GGAGVGGQQQQQQQ---GGAGVGGQQQQQ--KQSKK 370
BL_SaFr  302 VAGGQQQQQQQGGAGVGGQQQQQQQQQQGGAGVGGQQQQQQQQQ-GGAGVGGQQQQQQQKQSKK 364
BL_Culp  299 VAGGQQQQQQQGGAGVGGQQQQQQQQQ-GGAGVGGQQQQQQQQQQGGAGVGGQQQQQQQKQSKK 361

HL_SoFl  371 VVKTVTTKVVTTKKQKQQQQQQQQAGAVVGGQQQQQQQQQQAGAVVGGQQQQQQQHYLAGYRTA 434
BL_SaFr  365 VVKTVTTKVVTTKKQKQQQQQQQ-AGAVVGGQQQQQQQQ----------------HHLAGYRTA 411
BL_Culp  362 VVKTVTTKVVTTKKQKQQQQQQ--AGAVVGGQQQQQQQQ----------------HHLAGYRTA 407

HL_SoFl  435 APVYTGYAGRYAGAVAGAAAGARGLVY 461
BL_SaFr  412 APVYTGYAGRYAGAVAGAAAGARGLVY 438
BL_Culp  408 APVYTGYAGRYVGAVAGAAAGARGLVY 434
```

Figure S6B

```
HL_SoFl     1 MYFKTSVLVLVAATFVASYPMHGSYGGWGSQGQSQSQAQGQMMGGHYGGMGQGQAQAQSQ  60
BL_SaFr     1 MYFKTSVLVLVAATFVASYPMHGSYGGWGSQGQSQSQAQGQMMGGHYGGMGQGQAQAQSQ  60
BL_Culp     1 MYFKTSVLVLVAATFVASYPMHGSYGGWGSQGQSQSQAQGQMMGGHYGGMGQGQAQAQSQ  60

HL_SoFl    61 GQMMGGHYGGMGQAQAQAQ---------------------AQALQAAQAAQVAQAQAQAQ  99
BL_SaFr    61 GQMMGGHYGGMGQGQAQAQSQGQMMGGYYGGMGQAQAQAQAQALQAAQAAQVAQAQAQA- 119
BL_Culp    61 GQMMGGHYGGMGQGQAQAQSQGQMMGGYYGGMGQAQAQAQAQALQAAQAAQVAQAQAQAQ 120

HL_SoFl   100 VQAQALKAAQAQAQAQAQAVEAAQAQAQAHAQAVQVAQAQAQAQA-QAAQAQA-----QA 153
BL_SaFr   120 -----LKAAQAQAQAQAQAVEAAQAQAQAQAQAVQVAQAQAQAQAVQAAQAQAQAAQAQA 174
BL_Culp   121 VQAQALKAAQAQAQAQAQAVEAAQAQAQAHAQAVQVAQAQAQAQA-QAAQAQAQA---QA 176

HL_SoFl   154 ARVVGSRRVVSVMRGMGGMSQSFTSGFGDGFATGYGKGVQTVQGVGSNGVVAFYPSVAMA 213
BL_SaFr   175 ARVVGSRRVVSVMRGMGGMSQSFTSGFGDGFATGYGKGVQTVQGVGSNGVVAFYPSVAMA 234
BL_Culp   177 ARVVGSRRVVSVMRGMGGMSQSFTSGFGDGFATGYGKGVQTVQGVGSNGVVAFYPSVAMA 236

HL_SoFl   214 FPTVASAGPSVVQEPVVEQVYEVVDGPVVSGVGAMAGAAAGAGCGGVGAGAAAAAGAGGV 294
BL_SaFr   235 FPTVASAGPSVVQEPVVEQVYEVVDGPVVSGVGAMAGAAAGAGFGGVGAGAAAAAGAGGV 294
BL_Culp   237 FPTVASAGPSVVQEPVVEQVYEVVDGPVVSGVGAMAGAAAGAGFGGVGAGAAAAAGAGGV 296

HL_SoFl   295 GGGAGAAAAG-SGVGAGAAAAAAAGGVEAEDEAEAEAKAEAEAEAEVEGEETVVAESGAA 332
BL_SaFr   295 GGGAGAAAAAGSGVGAGAAAAAAAGGVEAEDEAEAEAKAEAEAEAEVEGEETVVAESGAA 354
BL_Culp   297 GGGAGAAAAAGSGVGAGAAAAAAAGGVEAEDEDE----AEAEAEAEVEGEETVVAESGAA 352

HL_SoFl   333 AGAAAGASAGVVGTGFHAVPATMAYLPVMTVPHVATVAGGQQQQQQQQGGAGGQQQQQQQ 392
BL_SaFr   355 AGAAAGASAGVVGTGFHAVPATMAYLPVMTVPHVATVAGGQQQQQQQ-GGAGGQQQQQQQ 413
BL_Culp   353 AGAAAGAAAGVVGTGFHAVPATMAYLPVMTVPHVATVAGGQQQQQQQ-GGAGGQQQQQQQ 411

HL_SoFl   393 Q-GGARVGGQQQQQQQEGGAGVRGQQQQQQQQQQGGAGVGGQQQQQQQKQSRKVVKTVTT 451
BL_SaFr   414 QQGGAGVGGQQQQQ--EGGAG--GQQQQQQ----GKAGVGG-QQQQQQKQSKKVVKTVTT 464
BL_Culp   412 QQGGAGVGGQQQQQ--EGGAG--GQQQQQQ----GKAGVGG-QQQQQQKQSKKVVKTVTT 462

HL_SoFl   452 KVVTTTKQKQKQKQQQQAEAGGQQQQQQEGGAGAGGQQQQQQQEGGAGGQQQQQQQ-GGA 510
BL_SaFr   465 KVVTTTKQKQKQKQQ--AGAGGQQQQQQQAGAGVGGQQQQQQQEGGAGGQQQQQQQQGGA 521
BL_Culp   463 KVVTTTKQKQKQKQQ--AGAGGQQQQQQQAGAGVGGQQQQQQQEGGAGGQQQQQQQQGGA 519

HL_SoFl   511 GGVVGGVVPVAAAPVAAAPVYHGGYTGGFGGAAAGAAASAVSKSGSVVY 559
BL_SaFr   522 GGVVGGVVPVAAAPVVAAPVYHGGYTGGFGGAAASAAASAVSKSGSVVY 571
BL_Culp   520 GGVVGGVVPVAAAPVVAAPVYHGGYTGGFGGAAASAAASAVSKSGSVVY 569
```
